# The ratio of single to co‐colonization is key to complexity in interacting systems with multiple strains

**DOI:** 10.1002/ece3.7259

**Published:** 2021-06-06

**Authors:** Erida Gjini, Sten Madec

**Affiliations:** ^1^ Instituto Gulbenkian de Ciência Oeiras Portugal; ^2^ Center for Computational and Stochastic Mathematics Instituto Superior Técnico University of Lisbon Lisbon Portugal; ^3^ Institut Denis Poisson University of Tours Tours France

**Keywords:** co‐colonization, complexity, cooperation‐competition, evolution, multi‐species dynamics, single‐to‐coinfection ratio, social interactions, stress gradient hypothesis

## Abstract

The high number and diversity of microbial strains circulating in host populations have motivated extensive research on the mechanisms that maintain biodiversity. However, much of this work focuses on strain‐specific and cross‐immunity interactions. Another less explored mode of pairwise interaction is via altered susceptibilities to co‐colonization in hosts already colonized by one strain. Diversity in such interaction coefficients enables strains to create dynamically their niches for growth and persistence, and “engineer” their common environment. How such a network of interactions with others mediates collective coexistence remains puzzling analytically and computationally difficult to simulate. Furthermore, the gradients modulating stability‐complexity regimes in such multi‐player endemic systems remain poorly understood. In a recent study (Madec & Gjini, *Bulletin of Mathematical Biology*, 82), we obtained an analytic representation for *N*‐type coexistence in an SIS epidemiological model with co‐colonization. We mapped multi‐strain dynamics to a replicator equation using timescale separation. Here, we examine what drives coexistence regimes in such co‐colonization system. We find the ratio of single to co‐colonization, *µ*, critically determines the type of equilibrium and number of coexisting strains, and encodes a trade‐off between overall transmission intensity *R*_0_ and mean interaction coefficient in strain space, *k*. Preserving a given coexistence regime, under fixed trait variation, requires balancing between higher mean competition in favorable environments, and higher cooperation in harsher environments, and is consistent with the stress gradient hypothesis. Multi‐strain coexistence tends to steady‐state attractors for small *µ*, whereas as *µ* increases, dynamics tend to more complex attractors. Following strain frequencies, evolutionary dynamics in the system also display contrasting patterns with *µ*, interpolating between multi‐stable and fluctuating selection for cooperation and mean invasion fitness, in the two extremes. This co‐colonization framework could be applied more generally, to study invariant principles in collective coexistence, and to quantify how critical shifts in community dynamics get potentiated by mean‐field and environmental gradients.

## Graphical abstract

Multi‐strain interaction networks are complex and hard to understand. Here, we study epidemiological interactions between *N* microbial strains, generated from varying susceptibilities to co‐colonization. Analyzing how collective dynamics emerge from the full structure of the pairwise interaction matrix, we uncover the critical driver μ shifting coexistence regimes and stability‐complexity in these multi‐strain endemic systems. The insights obtained may apply more broadly to other multispecies eco‐evolutionary dynamics.

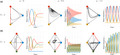

## 1 INTRODUCTION

Rich ecosystems comprise many species interacting together in a myriad of ways and on multiple temporal and spatial scales. Understanding the scope and consequences of such interactions has been the focus of countless theoretical ecology studies, starting with the seminal work by Lotka (1926) and Volterra (1926) on mathematical models of the population dynamics of interacting species. This model has been later extended and sophisticated by many other theoretical studies (May 1972; Pascual et al., 2006), and is currently extensively used to characterize interaction networks in empirical microbiome communities (Bucci et al., 2016; Stein et al., 2013). Theoretically, a crucial question has been to study stability and coexistence patterns in such Lotka–Volterra multi‐species communities, analyzing both structured ecological networks and random networks (Serván et al., 2018; Song & Saavedra, 2018). Modeling efforts seek to understand organizing principles for species composition, including the balance between competition and cooperation (Mougi & Kondoh, 2012).

Overall, analysis of such models with arbitrarily high dimensionality has been and continues to remain difficult. In particular, beyond the complexity‐stability debate which represents a major force in ecology (Landi et al., 2018; May 1972; McCann, 2000), many studies are increasingly addressing the problem of deriving collective dynamics from pairwise outcomes between species, which constitutes another major challenge, both at an analytical (Levine et al., 2017; Momeni et al., 2017) and empirical (Friedman et al., 2017) level.

The challenge of high‐dimensionality in ecological microbial networks parallels a similar challenge in the epidemiology of polymorphic pathogen systems, where understanding the mechanisms and forces that maintain diversity among interacting strains, is also an area of active research (Cobey & Lipsitch, 2012; Gupta & Anderson, 1999; Lipsitch et al., 2009; Wearing & Rohani, 2006). While it is well recognized that population patterns of infection are to a large extent determined by susceptibility to infection, most multi‐strain SIR models, inspired from influenza, dengue, and malaria parasites, have focused on cross‐immunity between strains as driver of population structure (Gog & Grenfell, 2002; Gomes et al., 2002; Gupta et al., 1998; Lin et al., 1999). Yet, other factors, besides persistent host immunity, may make strains compete or cooperate with each other, and it remains unclear which environmental variables also contribute to their epidemiologic fitness.

Here, we bridge between multi‐species ecology and multi‐strain epidemiology, revisiting coexistence and diversity in a new context. We explore another mode of strain interactions, namely altered susceptibilities to coinfection, whereby *N* strains compete in SIS endemic scenarios of no persistent immunity and no virulence. Coinfection models with up to two strains have described vulnerability to coinfection with a single parameter (Alizon et al., 2013; Davies et al., 2019; Gaivão et al., 2017; van Baalen & Sabelis, 1995), two coefficients (Lipsitch, 1997) or four coefficients (Gjini et al., 2016) depending on model structure and aims, but very few analytical investigations have been done for a larger number of interacting strains (Adler & Brunet, 1991), recognizing the difficulties of including within‐and between‐strain details for such coefficients (Mosquera & Adler, 1998). Moreover, analytic solutions for strain frequency dynamics in coinfection models remain rare, due to nonlinearities even for *N* = 2.

In a recent co‐colonization (coinfection) SIS model framework, with *N*‐strains, we have simplified the complex ecology embedded in *N* ^2^ epidemiological variables (Madec & Gjini, 2020). Using timescale separation, we obtained a model reduction from the matrix of pairwise coinfection vulnerabilities between strains. This coincides with a special replicator equation (Cressman & Tao, 2014; Hofbauer & Sigmund, 2003) by which we can predict explicitly multi‐strain frequency evolution. This *N*‐dimensional model reduction makes the entire epidemiology more accessible to analysis, and relates emergent collective dynamics to the ensemble of pairwise competitive outcomes, not only qualitatively but moreover in an explicit quantitative manner.

In the present article, we harness the simplicity of this co‐colonization model framework (Madec & Gjini, 2020) to investigate coexistence, stability, and evolution of such multi‐strain systems with variable co‐colonization susceptibility coefficients among strains. We start by studying the behavior of the system for different global variables such as total transmission intensity *R*_0_ and mean interaction coefficient in the pool of available strains *k*. We then study coexistence through random co‐colonization interactions, where the matrix coefficients are drawn from fixed distributions, and can range from competitive to cooperative links. We ask what is the number of strains that can coexist when starting from a pool of *N* strains, and in which diversity–stability configuration. We uncover rich transient and asymptotic behavior of such systems, where steady states, limit cycles, multi‐stability, and chaotic attractors are possible.

We find that the ratio of single‐ to co‐colonization is a critical factor in collective dynamics, by modulating the asymmetry in pairwise invasion fitness between types, and consequently, the dynamic complexity of the system as a whole. This ratio is key to observe the emergent context dependence of strain interactions (Bascompte, 2019; Coyte & Rakoff‐Nahoum, 2019) in our model. We argue that the analytically explicit form of this ratio in our formalism enables direct connection with the stress gradient hypothesis (SGH) in ecology (Bertness & Callaway, 1994; Callaway & Walker, 1997). This hypothesis postulates that as stress increases, the importance of positive facilitative effects increases in a community, whereas in benign environmental conditions, competitive effects are higher; a finding that emerges also from our results. In support of complex higher‐order dynamics emergent from simple pairwise interactions, with critical links between mean and variance, we uncover the exact formulation for why the sum as a collective is much more than its parts. Our results invite a deeper understanding of the biology of endemic multi‐strain systems and point to key global modulators of collective polymorphic coexistence in nature.

## 2 METHODS

### 2.1 *N*‐strain SIS model with co‐colonization

We study the epidemiological model (Madec & Gjini, 2020) given by the following ordinary differential equations:

(1) $$\left\{\begin{array}{cc} \dot{S}=m\left(1‐S\right)‐S\sum_{j=1}^{N}F_{j} & \\ {\dot{I}}_{i}=F_{i}S‐mI_{i}‐I_{i}\sum_{j}K_{\mathrm{ij}}F_{j}, & \;1\leq i\leq N\\ {\dot{I}}_{\mathrm{ij}}=I_{i}K_{\mathrm{ij}}F_{j}‐mI_{\mathrm{ij}}, & \;1\leq i,j\leq N \end{array},\right)$$

where $F_{i}=\beta \left(I_{i}+\sum_{j=1}^{N}\frac{1}{2}\left(I_{\mathrm{ij}}+I_{\mathrm{ji}}\right)\right)$ gives the force of infection of strain i, and the variables S, $I_{i}$ and $I_{\mathrm{ij}}$ refer to the proportion of susceptible hosts, singly colonized by strain i and co‐colonized by strains i and j. Notice that $S=1‐\sum I_{i}+I_{\mathrm{ij}}$, thus the dimension of the system is effectively N+N(N‐1)/2. A key assumption in the model is that hosts co‐colonized with different strains i and j transmit either with equal probability. In the above notation, m=γ+r, is the infected host turnover rate, encapsulating both clearance rate γ of colonization episodes and recruitment rate of susceptible hosts r (balanced by natural mortality rate r=d).

This model has also been described in detail previously (Gjini & Madec, 2017) for the case of N=2. The model allows for each strain to interact differently with other strains upon co‐colonization, altering the susceptibility of an already‐colonized host to incoming strains. The magnitude and type of such interactions are described by the matrix K, where values $K_{\mathrm{ij}}$ above 1 indicate facilitation between strains, and values of $K_{\mathrm{ij}}$ below 1 indicate inhibition or competition between strains. We do not make any specific assumptions on the mechanisms underlying such interactions, but a key feature of this formulation is the explicit incorporation of intra‐strain and inter‐strain interactions. The derivation of the slow‐fast dynamics decomposition (Madec & Gjini, 2020) lies on the assumption that the variance of such interaction coefficients is low, thus deviation from neutrality of $K_{\mathrm{ij}}$, with respect to a reference k, is small. In our particular simulations here, we assume a normal distribution for such interaction coefficients. We can write every $K_{\mathrm{ij}}$ as: $K_{\mathrm{ij}}=k+\varepsilon \alpha_{\mathrm{ij}}$ under the assumption that ε is small.

Although many different parametrizations are possible, we define $k=\left(\sum_{1\leq i,j\leq N}K_{\mathrm{ij}}\right)/N^{2}$ as the statistical mean of the co‐colonization interaction coefficients between all possible strains in the available pool. We define the deviation from neutrality ε as the standard deviation of (*K_ij_*)_1 ≤_ *_i_*_,_ *_j_*_ ≤_ *_N_*. This leads to the normalized interaction matrix

$$A=\left(\alpha_{\mathrm{ij}}\right)=\frac{K_{\mathrm{ij}}‐k}{\varepsilon },$$

to have the same distribution as K, but with mean 0 and variance 1, at the start of dynamics in the unpruned community. We adopt analysis and simulations to understand the behavior of the epidemiological model for different assumptions on the co‐colonization interaction matrix, and for different system size N. We use the reduced system (of N equations) for dynamics on the slow timescale εt (Madec & Gjini, 2020) to obtain the steady‐states of the system and analyze their properties and stability (see Box 1).

## 3 RESULTS

In the present model, the entire system is structured as a collection of hosts that can be in different colonization states: susceptible, singly colonized, or co‐colonized. We consider a multi‐type infection, transmitted via direct contact, following susceptible‐infected‐susceptible (SIS) epidemiological dynamics with co‐colonization (Madec & Gjini, 2020). Adopting a general formulation for diversity, we assume there are *N* types, without specifying the mechanisms for their definition. Thus, with an ordinary differential equations model (see Section 2), we describe the proportion of hosts in several compartments: susceptibles, *S*, hosts colonized by one type *I_i_*, and co‐colonized hosts *I_ij_*, with two types of each combination, independent of the order of their acquisition. The model structure follows that of Gjini et al. (2016); van Baalen and Sabelis (1995) allowing also for same strain coinfection (*I_ii_*). Hosts in the mixed coinfection compartment (*I_ij_*) transmit either strain with equal probability. Fitness differences in this system are encoded in how strains interact with each other upon co‐colonization (*K_ij_*), whether there is facilitation between resident and co‐colonizer (*K_ij_* > 1) or inhibition (*K_ij_* < 1), and its exact magnitude, – assuming equivalence in transmission *β* and clearance rate *γ*. The structure of the *K_ij_*, subject to small deviation from perfect symmetry, is central to multi‐strain dynamics. We take no account of other biological details such as mutation, seasonality in transmission, or heterogeneity in the host population, which may influence the transmission dynamics of particular strains. These exclusions serve our purpose to assess the impact of selection imposed by co‐colonization interactions between given strains in the host population on temporal trends in individual strain frequencies.

In an earlier mathematical investigation (Madec & Gjini, 2020), we have derived in detail two timescales in this multi‐strain system: a *fast* one, given by the neutral model, and a *slow* one governed by the variation in co‐colonization coefficients *K_ij_*. During fast dynamics, the system stabilizes conserved quantities such as the endemic prevalence of single and co‐colonization whereas over the slow timescale, strain selective dynamics unfold (see Box 1).

BOX 1 **Key features of the *N*‐strain co‐colonization model (Madec & Gjini, 2020)**

**Deviation from symmetry in interaction trait space: the basis of the framework**

We conceptualize each altered susceptibility to co‐colonization (i.e., interaction coefficient) between closely related strains, as a mean value *k* plus some deviation from symmetry, thus re‐writing it as:

(2) $$K_{\mathrm{ij}}=k+\varepsilon \alpha_{\mathrm{ij}},$$

where 0<ε<1 is small. Thus, $A=(\alpha_{\mathrm{ij}})$ is the normalized interaction matrix, relative to the reference k. The parameter k in this case encodes mean interaction in co‐colonization between any two strains, which, if k>1 describes mutual facilitation, and if k<1 describes mutual inhibition. Another key epidemiological parameter is the basic reproduction number, $R_{0}$, in our model given by β/m (Section 2), describing the intensity of transmission, and defined as the average number of secondary infections that arise from a single case in a naive population (Diekmann et al., 1990).

**Fast and slow dynamics: derivation of the replicator equation in a new context**

On the fast time‐scale (ε=0), strains behave as neutral ($K_{\mathrm{ij}}\equiv k$). The system reaches the central manifold where total prevalence of susceptibles S, total prevalence of single colonization I, and total prevalence of co‐colonization D are conserved, and depend only on mean epidemiological parameters:

(3) $$S=\frac{1}{R_{0}},\;I=\sum_{i=1}^{N}I_{i}^{*}=\frac{R_{0}‐1}{R_{0}+R_{0}k\left(R_{0}‐1\right)},\;D=\sum_{1\leq i,j\leq N}I_{i,j}^{*}=\frac{1}{\left(R_{0}‐1\right)k}I$$

but, where each individual strain frequency is neutrally stable and free to vary $z_{i}\in \left[0,1\right]$. On the slow time scale τ=εt, consistent with weak selection, exact asymmetries between strains play out, and under the conservation law above, individual frequencies $z_{i}$ obey deterministic slow dynamics, given by an N‐dimensional replicator equation:

(4) $$\frac{d}{d\tau }z_{i}=\Theta z_{i}\cdot \left(\sum_{j\neq i}\lambda_{i}^{j}z_{j}‐Q\left(z\right)\right),\;i=1,\cdots ,N,\;\sum_{i=1}^{N}z_{i}=1,$$

where $Q\left(z\right)={\sum \sum }_{1\leq k\neq j\leq N}\;\lambda_{j}^{k}z_{j}z_{k}$, is a quadratic term symmetric on all strains, encapsulating the effect of the system as a whole on each individual strain. The quantity $\sum_{i}z_{i}=1$ is conserved and the rate Θ is given by: $\Theta =\beta \frac{\left(1‐S\right)\mathrm{ID}}{2{\left(1‐S\right)}^{2}‐\mathrm{ID}}$. This replicator equation links directly epidemiological SIS dynamics to Lotka–Volterra systems (Bomze, 1995) and multi‐strain co‐colonization processes to ecology and evolution (Nowak & Sigmund, 2004).

**From pairs to collective dynamics**

The above equations drastically reduce the system from N(N‐1)/2+N to N dimensions. Furthermore $z_{i}$ dynamics are a direct function of pairwise invasion fitnesses $\lambda_{i}^{j}$ between strains, which, for each pair (i,j), have the form:

(5) $$\lambda_{i}^{j}=\alpha_{\mathrm{ji}}‐\alpha_{\mathrm{jj}}‐\mu \left(\alpha_{\mathrm{ij}}‐\alpha_{\mathrm{ji}}\right).$$

The quantities $\lambda_{i}^{j}$ denote the initial rate of growth of strain i in an exclusion equilibrium where only strain j is resident, and depend on the ratio between single and co‐colonization μ which is given by

(6) $$\mu =\frac{I}{D}=\frac{1}{(R_{0}‐1)k}.$$

**Invariant principles in nonequilibrium multi‐strain dynamics**

On the slow timescale, at all times, and for all strains, the following relationships hold:

(7) $$z_{i}=\frac{I_{i}}{I}=\frac{D_{i}}{D},$$

stating that $z_{i}$, as a measure of dominance of strain i in the total prevalence of carriage, occupies an equal relative frequency in single ($I_{i}$) and co‐colonization ($D_{i}=\sum_{j}\left(I_{\mathrm{ij}}+I_{\mathrm{ji}}\right)/2)$). After solving for strain frequencies $z_{i}$, the Equation (4) can inform the epidemiological variables of the original system (1) as follows:

(8) $$I_{i}\left(t\right)=Iz_{i}\left(\tau \right);\;I_{\mathrm{ij}}\left(t\right):=Dz_{i}\left(\tau \right)z_{j}\left(\tau \right)$$

### 3.1 How mean‐field parameters determine type and tempo of selection

#### 3.1.1 What type of coexistence?

According to this model, global mean‐field parameters, such as those affecting transmission rate, β, basic reproduction number $R_{0}$, and mean interaction coefficient k, explicitly impact the multi‐strain dynamics over long time scales (εt). The equation for strain frequencies $z_{i}$ contains information for how they set the speed (Θ) and mode of strain stabilization (μ). More specifically, the relative dominance of single colonization in the system μ appears as a factor in pairwise invasion fitness ($\lambda_{i}^{j}=\alpha_{\mathrm{ji}}‐\alpha_{\mathrm{jj}}‐\mu \left(\alpha_{\mathrm{ij}}‐\alpha_{\mathrm{ji}}\right)$) of i when invading a j‐ only equilibrium. The μ parameter modulates the importance of cross‐strain asymmetry in co‐colonization trait comparison between two strains. The higher μ is, the higher the prevalence of single colonization, thus the more important the asymmetry between how i and j invest in the mixed co‐colonization compartment $I_{\mathrm{ij}}$. Because $I_{\mathrm{ij}}$ hosts transmit both i and j with equal probability, the relative superiority of i will be reduced from such “altruistic” investment; thus, it appears with a negative sign $‐\mu (\alpha_{\mathrm{ij}}‐\alpha_{\mathrm{ji}})$ in invasion fitness. While this component of invasion fitness of i is sensitive to feedbacks from global transmission and mean parameters between strains, the other fitness component of i in $\lambda_{i}^{j}$ depends only on characteristics of the resident, namely on the relative strength of inter‐ versus intra‐ strain interaction of the resident ($\alpha_{\mathrm{ji}}‐\alpha_{\mathrm{jj}}$), which indicates how much the resident j promotes its own coinfection, compared to its vulnerability to coinfection by i.

**FIGURE 1:**
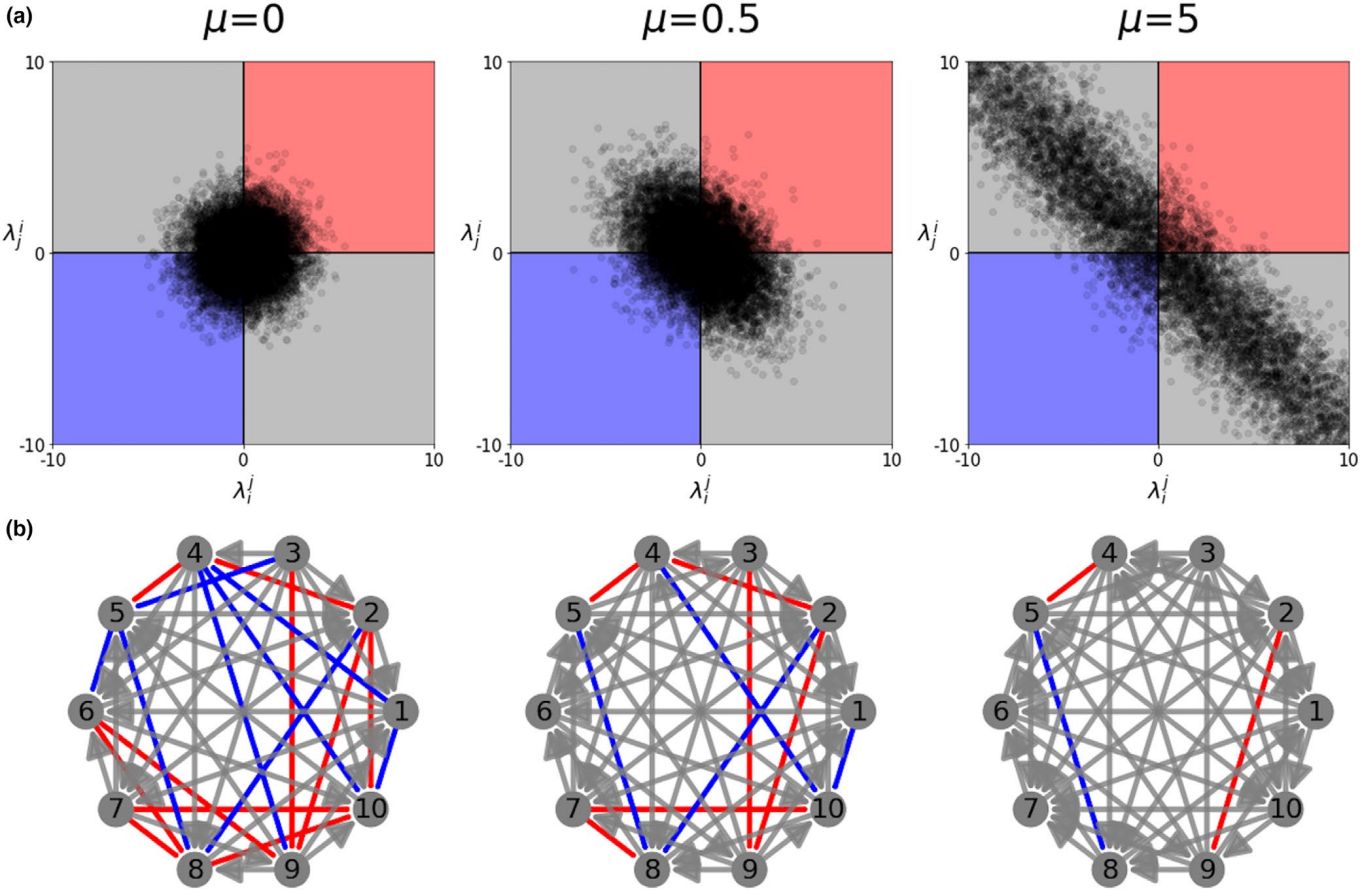
Collective coexistence from pairwise invasion fitnesses and the effect of single‐to co‐colonization ratio μ. (a) Effects of μ on the repartition of the pairwise invasion fitnesses. In our model, there are four possible outcomes between any two strains (depending on the values of $\lambda_{i}^{j}$ and $\lambda_{j}^{i}$): extinction of i, extinction of j, coexistence or bistability of the exclusion equilibria, the latter being known also as priority effects (Ke & Letten, 2018). The coefficients $\alpha_{\mathrm{ij}}$ of the matrix $A=(\alpha_{\mathrm{ij}})$ are randomly generated from a normal distribution N(0,1). When μ→0, the partitioning (probabilities) between the four outcomes is the same. When μ increases, the strain pairs, for the same A matrix, follow more likely competitive exclusion dynamics. Among 10,000 random simulations of *A*, as we increase μ, we find 51% of pairwise competitive exclusion for μ=0, 64% exclusion for μ=0.5 and 92% exclusion for μ=5. (b) Effect of μ on the multi‐strain invasion (pairwise λ) networks. Red line: coexistence, blue line: bistability, and a gray arrow indicates competitive exclusion (the arrow points to the winner between the two). Here we fixed the normalized interaction coefficients between strains (matrix A) and N=10 strains, and we varied μ. As μ increases even though the actual normalized interaction coefficients remain fixed, the effective outcomes between each pair of strains, being μ‐dependent, tend to exclusion for $\lim_{\mu \to +\infty }\lambda_{i}^{j}+\lambda_{j}^{i}=0$, with the gray edges becoming more common

Notice, that for fixed normalized variation among strains, hence rescaled matrix $A=(\alpha_{\mathrm{ij}})$, increasing μ in the system, amplifies asymmetries among all pairs of strains. Mathematically this is reflected in the correlation between $\lambda_{i}^{j}$ and $\lambda_{j}^{i}$ tending to ‐1, which leads to more pairwise competitive exclusion. We illustrate this for the case of N=2 in Figure 1a, where for randomly generated rescaled interaction matrices A (Normal distribution with mean 0 and variance 1), increasing μ stretches the region occupied by $\lambda_{i}^{j}$ towards the competitive exclusion zone ($\lambda_{i}^{j}>0$, and $\lambda_{j}^{i}<0$ and viceversa). This phenomenon applies also to the case of higher N, where effectively the multi‐strain network is derived from pairwise invasion fitnesses between any two constituent strains (Figure 1b). As the ratio of single to co‐colonization, μ, increases, the proportion of edges between constituent strains, that lead to competitive exclusion increases, making collective coexistence more probable as a result.

**FIGURE 2:**
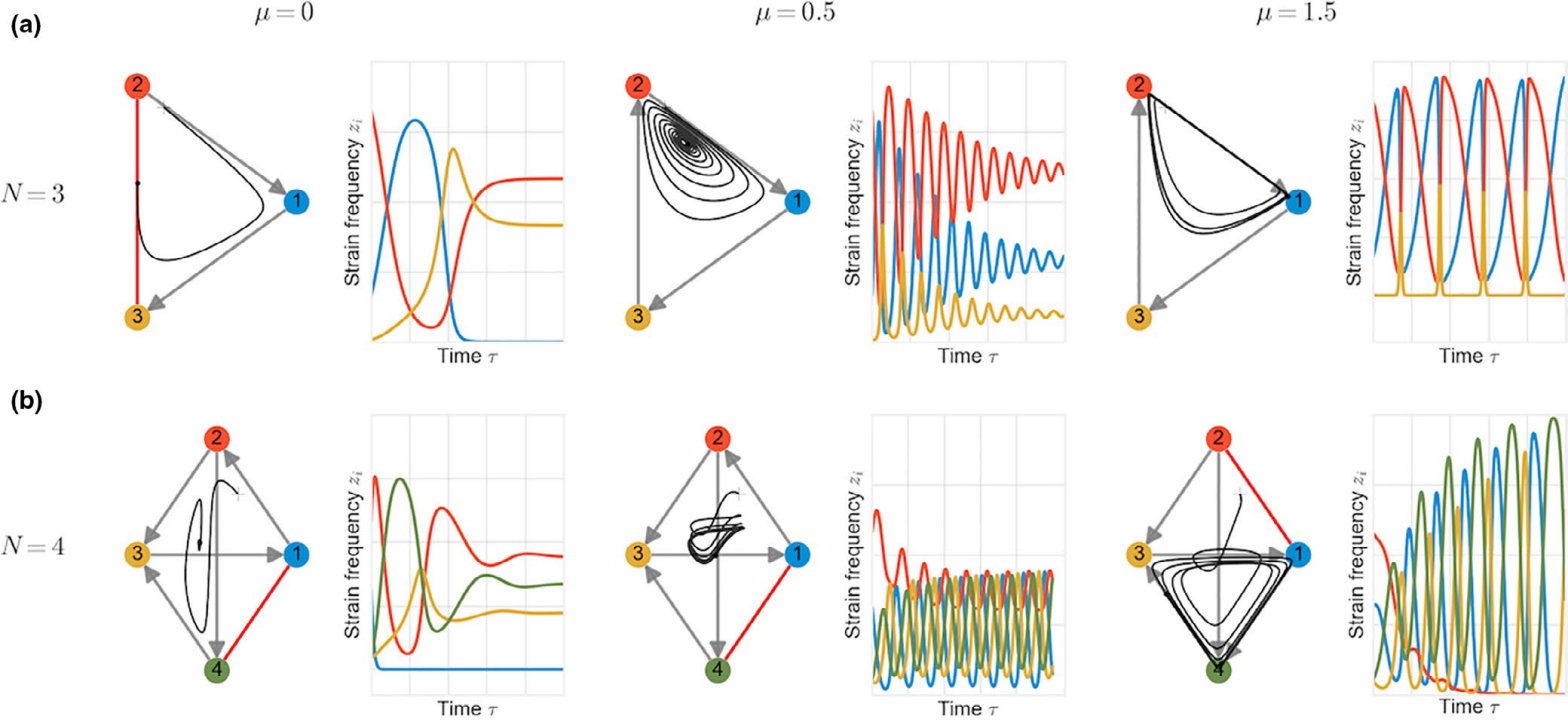
Increasing the ratio of single to co‐colonization (μ) increases the complexity of multi‐strain dynamics. We illustrate two examples with fixed interaction matrix A and shifting μ for two system sizes: (a) N=3 and (b) N=4. Here, we fixed the normalized interaction coefficients between strains (matrix A) and we varied μ from 0 to 0.5 and 1.5. Because μ affects the $\lambda_{i}^{j}$ (Equation 5), for each μ, we obtain a different effective competition network between strains modulated by top‐down factors. The plots show the strain frequency $\left(z_{i}\right)$ dynamics following the replicator equation on the slow manifold. For N=3 and N=4, for small value of μ closer to zero, the final attractor is a stable steady state, while for a larger value of μ, the stable attractor is a union of heteroclines of three strains (May Leonard type or rock‐paper‐scissors dynamics). For intermediate values of λ (μ=0.5) in this case we obtain a stable spiral of coexistence for N=3 and a limit cycle of coexistence for N=4, both consistent with an increase in rate of strain turnover

To illustrate concretely how the ratio μ impacts ecological coexistence, we visualize multi‐strain dynamics as a function of μ in Figure 2, for the case of N=3 and N=4. Under fixed interaction asymmetries A, changing the ratio between single and co‐colonization, shifts multi‐strain dynamics from stable coexistence towards limit cycles and ultimately towards heteroclinic cycle type oscillations, with effectively only one strain persisting for long periods of time, to be subsequently replaced by another one, and so on. We find that for μ→0 and random $\alpha_{\mathrm{ij}}$, we have a case of a general replicator equation whose steady‐state analysis when $\alpha_{\mathrm{ii}}=0$ may be reduced to Generalized Lotka–Volterra (GLV) dynamics with constant growth rates and random interactions. Whereas for the case of μ≫1, dynamics converge to hyper‐tournament dynamics studied by Allesina and Levine (2011) for $\alpha_{\mathrm{ij}}=\pm 1$ and Grilli, Barabás, et al. (2017) for the case in which $\alpha_{\mathrm{ij}}\neq \pm 1$. These extreme regimes of behavior are expected in the replicator equation for special cases, but the novelty in our framework is that we have identified a global system quantity, the ratio between single and co‐colonization, as a tuning parameter for moving our multi‐strain dynamics between such extremes (see Box 2).

BOX 2 **Single to co‐colonization ratio as a tuning parameter for complexity of attractors**

One limit for μ may be achieved by either increasing global facilitation k→∞, or increasing transmission intensity $R_{0}=\frac{\beta }{m}\to \infty$. The other limit can be achieved by increasing competition in co‐colonization or decreasing transmission intensity. Although in reality, these limits may never be literally attained biologically, here we describe the mathematical trends of the system, which will most likely reside in the intermediate range. For a more detailed analysis of system behavior and speed of the dynamics $\Theta_{\lim}$ in these limits, see also Text S2, and Figures S2 and S3. A specific example with dynamics close to these limits is provided in Figure S4.

**Limit 1: Co‐colonization, μ→0 ($R_{0}↑$ or k↑)**

**Quality of the dynamics**

Recall that $\lambda_{i}^{j}>0$ implies that the strain i (the invader) may invade the system with only strain j (the resident) present, starting from a very small frequency. In the limit μ→0, this pairwise invasion fitness reduces to:

$$\lambda_{i}^{j}=\alpha_{j,i}‐\alpha_{j,j}.$$

Importantly, in this limit, the fitness $\lambda_{i}^{j}$ depends only on the coefficients of the resident, where $\alpha_{j,i}$ measures how much the resident j contributes to mixed co‐colonization with strains i and j: $I_{\mathrm{ij}}$ (transmission and fitness of other strains), and $\alpha_{j,j}$ how much the resident j contributes to self co‐colonization: $I_{\mathrm{jj}}$ (transmission and fitness of itself). Hence, the relative fitness of the invader i depends only on the resident j. Invasion is possible if and only if $\alpha_{j,i}>\alpha_{j,j}$.

This phenomenon in a system of N players can increase the availability of independent niches. For example, if $\alpha_{j,j}>\alpha_{i,j}$ for all j=1,⋯N and i≠j then all strains may be stable residents when alone, leading to at least N stable monomorphic equilibria. Cycles or more complex dynamics are rare.

The final outcomes can vary because the structure of the matrix Λ is free in principle. In the special case $\alpha_{\mathrm{jj}}=0$ for all j, then $\Lambda =A^{T}$, and the dynamics could be richer, in line with generic replicator systems (Yoshino et al., 2008). In that special case, under random $\alpha_{\mathrm{ij}}$ i.i.d. symmetric about 0, the probability of having a feasible equilibrium $z^{*}>0$ is exactly $1/2^{n}$, where n is the number of coexisting strains (Morrison, 2013; Serván et al., 2018). In general, for random A, with no special structure, qualitatively, the system likely has many stable steady states wherein only few species coexist.

**Speed of the dynamics**

1. $\Theta_{\lim}=0$. If k→∞, we have $\Theta :\frac{m}{2k}\to 0$. For fixed population turnover (*m*: clearance and death rate), the selective dynamics happen very slowly, that is, the dynamics are effectively neutral. Similarly if $R_{0}\to \infty$ via lower m (m→0), the multi‐strain system stays close to neutrality.
2. $\Theta_{\lim}>0$. If $R_{0}\to \infty$, via increases in transmission rate, β→∞, then $\Theta \to \frac{m}{2k}>0$ which is far from zero. In this case, the model reduction captures well the real dynamics and the qualitative study of μ→0 is appropriate.

**Limit 2: Single colonization, μ→∞ ($R_{0}↓$ or k↓)**

**Quality of the dynamics**

Recall the matrix Λ is the matrix of all pairwise invasion fitnesses between N strains in the system. In this limit, we have generically ||Λ|| → +∞ and Θ→0. In order to keep a bounded matrix Λ, we rewrite the system as

(9) $${\dot{z}}_{i}=\mu \Theta z_{i}\left[{\left(\left(\mu^{‐1}\Lambda \right)z\right)}_{i}‐z^{t}\left(\mu^{‐1}\Lambda \right)z\right],$$

Then, the speed is given by μΘ and the qualitative behavior by $\mu^{‐1}\Lambda$. Notice that when μ→+∞, the matrix $\mu^{‐1}\Lambda \to A^{T}‐A.$

Importantly, this matrix is skew symmetric, for which there are known mathematical results of the replicator equation in zero‐sum games (see Allesina & Levine, 2011; Chawanya & Tokita, 2002; Fisher & Reeves, 1995). The quadratic term in Equation (9) is zero: $Q\left(z\right)=z^{T}\Lambda z=0$. The N‐strain dynamics reduce to:

(10) $${\dot{z}}_{i}=\Theta_{\lim}z_{i}\left[{\left(\left(A^{T}‐A\right)z\right)}_{i}\right].$$

There exists exactly one nonnegative linearly stable equilibrium (in particular multistability is impossible). In practice, similar to the classical Lotka–Volterra model, there is a one‐parameter family of limit cycles parametrized by initial conditions. This type of coexistence is not structurally stable and will be lost for a large but finite value of μ leading in general to heteroclinic limit cycles among strains.

Denoting n the number of strains coexisting, for this skew‐symmetric case, it has been shown that only an odd number of strains may coexist (see also Figures S1 and S5). The probability to observe n=k strains, out of a total pool of N is

$$P\left(n=k\right)=\left\{\begin{array}{cc} 0 & \text{if}\;k\;\text{is}\;\text{even}\\ \left(\begin{array}{c} N\\ k \end{array}\right)2^{1‐N} & \text{if}\;k\;\text{is}\;\text{odd} \end{array}\right).$$

In this case, at equilibrium $\Lambda z^{*}=0$, hence $z^{*}$ is an eigenvector associated with a zero eigenvalue. For a general skew‐symmetric matrix, a zero eigenvalue is expected only when its size is odd (see also Grilli, Barabás, et al., 2017).

**Speed of the dynamics**

1. $\Theta_{\lim}=0.$ If $R_{0}\to 1$ decreases transmission intensity, either via lower β or higher m, then Θ→0, and selective dynamics are too slow, making the system effectively neutral.
2. $\Theta_{\lim}>0$. If k→0, $\mu \Theta \to \frac{m}{2}\left(R_{0}‐1\right)>0$. Thus dynamics are well defined and occur on a feasible timescale.

#### 3.1.2 How many strains coexist?

Using simulations with random strain interactions and varying μ, we find that the mean number of strains that can coexist (n) increases with μ (see full distributions in Figure S1 for N=10). In the limit μ→0 and with the additonal assumption that $\alpha_{\mathrm{ii}}=0$ for each i, the probability of a feasible (positive) steady‐state of n strains in our replicator equation is exactly the same as that of a positive steady state in GLV dynamics with equal growth rates and random interactions: $2^{‐n}$. As μ increases, the number of coexisting strains tends to N/2, with μ→∞ where N denotes the total pool size. In particular, when μ is low, lower numbers of coexisting strains are progressively more probable, but for large values of the ratio μ, favoring single to co‐colonization, stable coexistence tends to become restricted to only an odd number of strains, a feature expected in special cases of the replicator equation (Chawanya & Tokita, 2002). Such case of perfect pairwise exclusion between species has emerged as the best‐studied stabilizing competitive network involving intransitive competition among species, known as rock‐paper‐scissors tournament games (Allesina & Levine, 2011; Kerr et al., 2002). In our system, this extreme, obtained in the limit μ→∞, emerges as a special case of a far more general gradient mediated by different values of μ, where edges between any pairs of strains can vary among coexistence, exclusion and bistability, and with quantitative subtleties like in hypertournament games.

#### 3.1.3 How fast is the dynamics?

Thanks to the explicit formula for this critical ratio, one can immediately see that $\mu =I/D=1/k(R_{0}‐1)$ can change in two ways, either by changing basic reproduction number $R_{0}$, or by changing k, the mean interaction coefficient between strains in co‐colonization. If overall colonization increases ($R_{0}↑$), or if facilitation between strain increases (k↑), μ decreases, and viceversa: when total prevalence is lower, or strains compete more in co‐colonization, μ increases. So far, we have seen that the ratio μ determines completely the type of multi‐strain equilibrium, for a given standardized variation in strain interactions. But how fast is this equilibrium reached? This is determined by $\Theta =\beta \left(1‐\frac{1}{R_{0}}\right)\frac{\mu }{2{\left(\mu +1\right)}^{2}‐\mu }$, also explicit, in our replicator equation describing multi‐strain dynamics over the slow time εt. As μ changes, not only the selection “movie” changes, but also the effective speed at which such “movie” is played. Essentially, Θ decreases with μ, hence selection is slower with increases in $R_{0}$ or k. But there are in general three cases, illustrated in Figure S2. When changes in μ are obtained via $R_{0}$, if μ is small (i.e., $R_{0}$ large) it is possible that increasing μ increases Θ. This would be obtained either from lower β or higher m in $R_{0}=\beta /m$. In such case, reducing colonization prevalence from relatively high to lower levels (via lower strain transmission/growth, or faster host birth‐death, shorter colonization episodes) not only acts to increase the complexity of the dynamics, but also to speed up multi‐strain selection in the biological co‐colonization “game”.

### 3.2 Coexistence, stability, and diversity

The community dynamics resulting purely from co‐colonization interactions between strains can be very complex, and ranges from simple coexistence equilibria to limit cycles and even possible wildly oscillatory dynamics. Noting that dynamics among strains are bounded in our system, failure to identify existence of a steady state necessarily implies the existence of another long‐time attractor, of a more complex nature. We find the dimension of the attractor is 0 for small μ and becomes 1, and may even exceed 1, for larger values of this parameter. This is what we refer to as complex coexistence.

**FIGURE 3:**
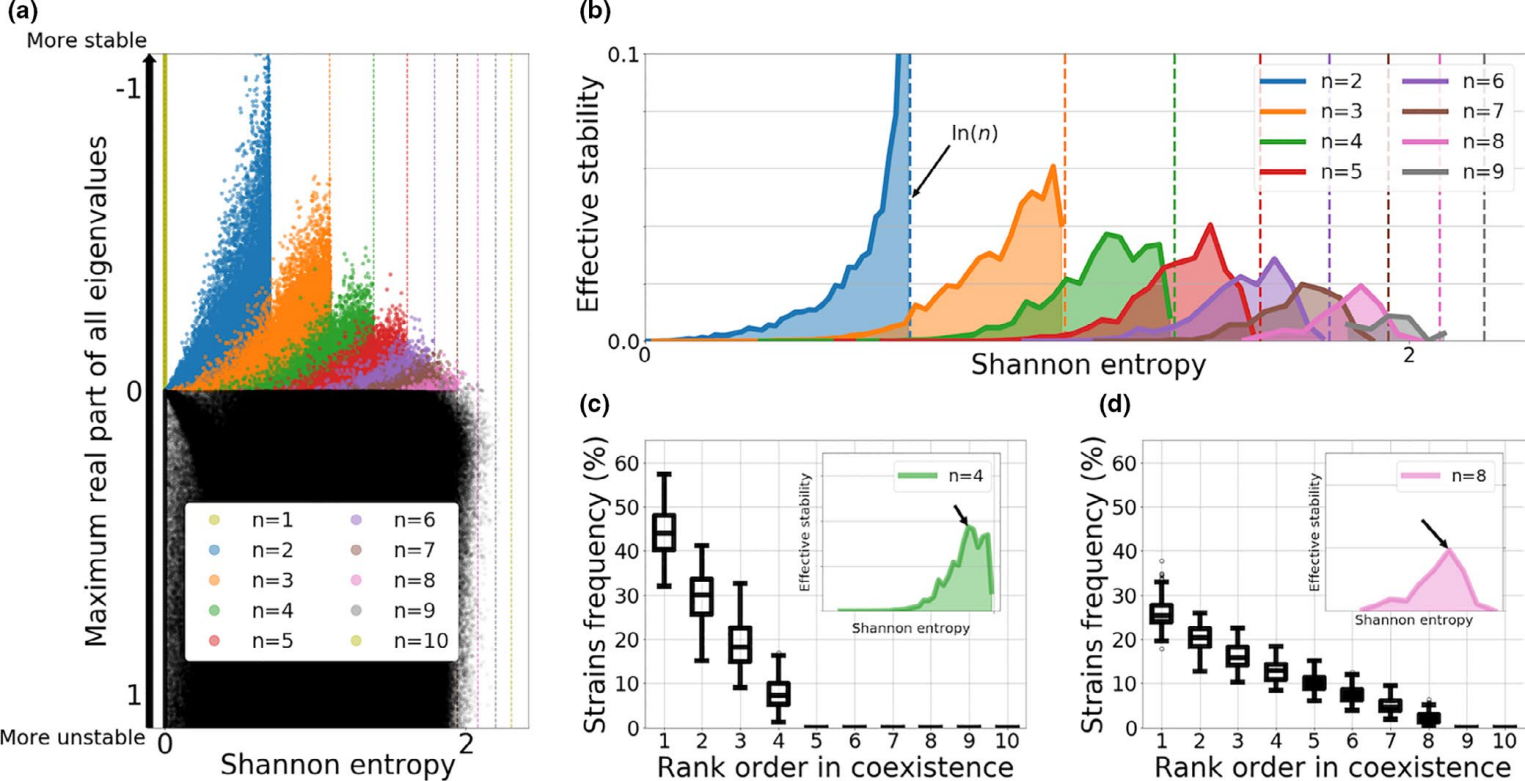
Diversity‐stability relationships for n‐strain coexistence starting with a pool of N=10 strains. (a) Summary of 100,000 simulations with a random normalized interaction matrix A, with each entry $\alpha_{\mathrm{ij}}$ drawn from the Normal distribution N(0,1). μ=0.05. The equilibria of each system are mapped as dots in this plot, after computing their local stability and Shannon entropy. (b) Effective stability is calculated as a product between the mean dominant eigenvalue and proportion of steady states at each entropy level, stratified by number of strains coexisting n. (c) Plot of the rank‐ order frequency distribution of strains at the “optimal intermediate evenness” for n=4 strains coexisting out of a pool of 10. (d) Plot of the rank‐ order frequency distribution of strains at the “optimal intermediate evenness” for n=8 strains coexisting out of a pool of 10

As the important debate in ecology about the relationship between stability and diversity in ecosystems is still ongoing (Landi et al., 2018; May, 1972; McCann, 2000; Odum & Barrett, 1971; Tilman & Downing, 1994), here we set out to examine this relationship in our system, assuming random interaction structure between strains, described in the matrix A, and fixing the total pool size of strains N. Thus, we study the model's rich behavior for a relatively larger pool of strains in the system, namely N=10 (Figure 3). We sample the normalized interaction matrix A randomly from a normal distribution N(0,1). For each interaction matrix, we compute numerically the feasible equilibria of the system (verifying $z_{i}\geq 0,\forall i\in \left\{1,2,..N\right\}$), and for each equilibrium found, we evaluate the number of strains coexisting, n, the local stability of the steady state (see Appendix), and its associated Shannon entropy $H=‐\sum_{i}^{N}z_{i}^{*}\ln\left(z_{i}^{*}\right),$ also known as species evenness in ecology. By simulating different randomly generated normalized interaction matrices A, we can explore regimes of exclusion, multi‐stability, multi‐strain coexistence, limit cycles and even chaos. In this analysis we use the definition of equilibrium stability (McCann, 2000), although there are also other notions of general stability related to permanence (Law & Morton, 1996) that could be informative.

Analyzing coexistence patterns under random allocation of co‐colonization interaction coefficients (Figure 3), there are multiple phenomena to note. First (and trivially) the higher the number of strains coexisting (n≤N), out of a pool of N strains, the higher the maximal Shannon entropy (evenness) in coexistence, delimited mathematically by $H=‐\sum_{i}^{n}\frac{1}{n}\ln\left(\frac{1}{n}\right)=\ln\left(n\right)$, a function that increases with n (dashed lines in Figure 3a). Second, within a given n, the higher the evenness in coexistence, the higher the stability of such steady‐state, as measured by the negative part of the dominant eigenvalue of the Jacobian matrix evaluated at that equilibrium, a result similar to Song and Saavedra (2018) using a Lotka–Volterra framework. In contrast, across different n, as maximal entropy increases, the maximal stability of coexistence steady states tends to decrease (color peaks decreasing diagonally down from left to right in Figure 3a). This implies that under randomly sampled interactions, hence in randomly assembled communities, stable coexistence equilibria with more strains, and more evenly distributed strain frequencies, become harder to obtain.

Next, when formally computing the probabilities of reaching a stable n‐strain coexistence in this model, we find that the relative probability of stable n‐strain steady‐state decreases with n, thus confirming that the more strains there are, randomly interacting, the harder it is for all of them to coexist (relative comparison among colored dots frequencies in Figure 3a). In contrast to the overall stable steady states (2%), there are many more unstable steady states in the system (98%), spread at any entropy (evenness) and stability level, with no apparent order or hierarchy (black dots in Figure 3a). This indicates that stable coexistence at a steady state is rare under random co‐colonization interactions, and that the attractors are often of dimension 1 or more. This also suggests that the number of coexisting strains in that case is not a limiting factor.

Quantification and stratification of these probabilities jointly by n, stability and evenness, combines the feasibility of a given state with its expected stability and strain composition. Then, to visualize in more detail this distribution, among steady states with a given n, for each entropy level E, we multiply the existence probability of a stable steady state (feasibility) f(E) with the absolute value of mean dominant eigenvalue (mean stability) of such steady state s(E) and plot the resulting *effective stability* =f(E)s(E) for each n (Figure 3b). This integrates two opposing forces: on one hand higher‐evenness equilibria are less feasible and harder to reach under random sampling of interaction coefficients from a pool of N strains, that is, f(E) decreases with E; on the other hand, more evenness among strains is associated with more stability in coexistence, s(E) increases with E, similar to (McCann, 2000). As can be seen in the emergent peaked curves in Figure 3b, such trade‐off between probability of stable coexistence and stability gives rise to an optimal intermediate evenness for n‐strain coexistence, for any n, where stable coexistence is sufficiently feasible in the first place, and secondly where the stability of that equilibrium to perturbations is sufficiently high. Thus, mean stability of n‐strain coexistence is effectively maximized at particular, typically intermediate, values of Shannon entropy (Figure 3c,d). In practice, independently of particular strain composition, out of a total pool of N, for a given number coexisting, n, the rank‐frequency distribution of strains should be preserved. Such “optimal” rank‐order frequency distribution will display higher variance for low number of coexisting strains (for example n=4), but be more robust for higher n (e.g., n=8).

In Figure S5, we explore the same phenomena for the case of a higher μ, tending to dominant single colonization (μ=10 more likely applies to a system like pneumococcus serotypes, Gjini et al., 2016). We find the ecological forces leading to an optimal effective stability at an intermediate entropy range remain robust also in this case. This limit also verifies that for the same randomization of interaction coefficients A, typically more strains coexist than for lower μ, and in particular, tending to an odd number of them (Chawanya & Tokita, 2002), while their joint dynamics become more complex (Box 2).

### 3.3 The role of the ratio μ in multi‐stability

**FIGURE 4:**
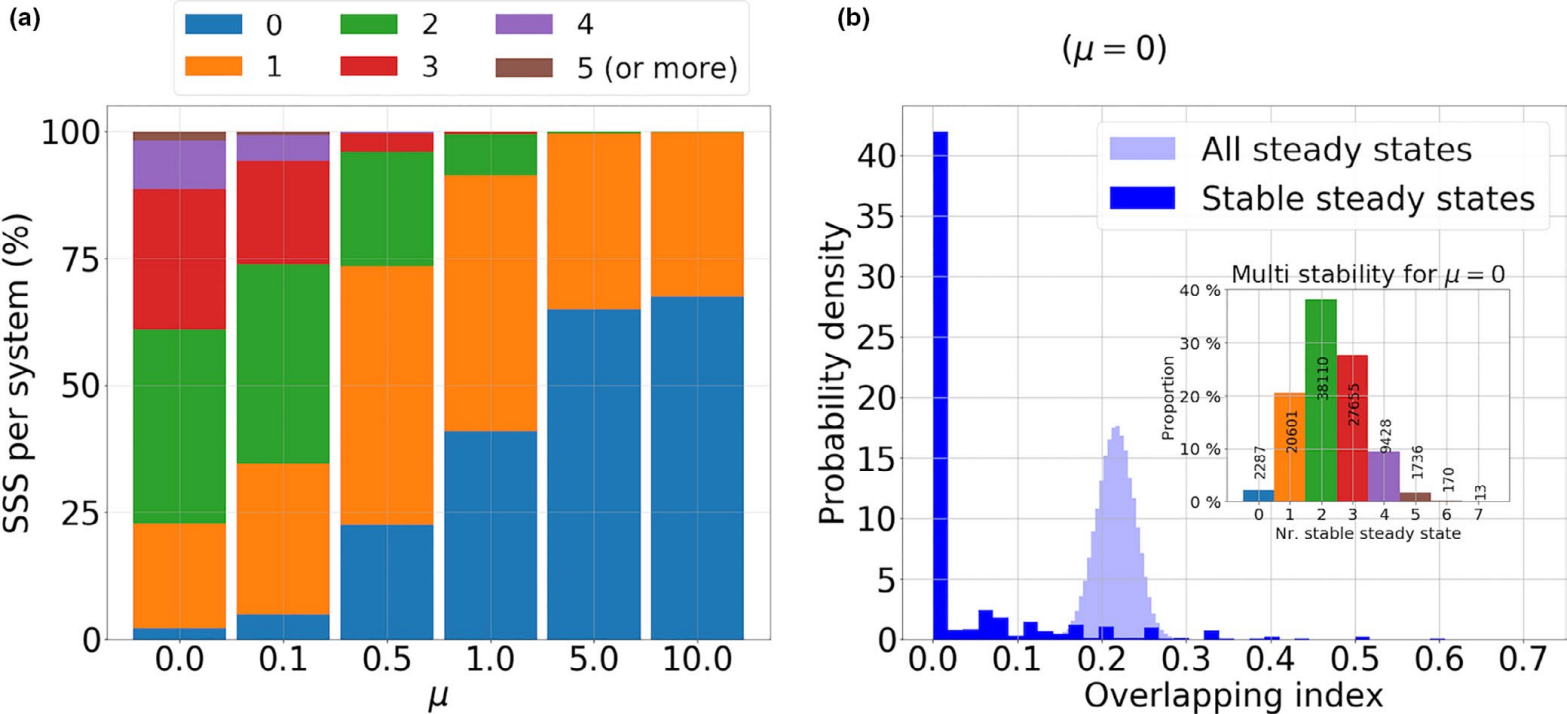
System complexity as a function of μ and multi‐stability. (a) For several values of μ, 100,000 systems of N=10 strains have been generated (using a normal distribution of A). For each value of μ, we show the proportion of systems with a given number of stable steady states (in {0,⋯,6}). As μ increases, the proportion of systems without any stable steady state increases, reflecting larger potential for complex coexistence dynamics, where the attractors may be limit cycles, heteroclinic cycle or even strange attractors. For μ<0.5 (high transmission or competition between strains), stable multi‐strain coexistence is very probable, and most of the systems have several stable steady states, thus multi‐stability is common. (b) In the particular limit case of μ=0, and for each of the 100,000 systems, we compare the strain overlapping index SOI (see Appendix) within *all* steady states and within only the *stable* steady states. We see that without the constraint of stability, two steady states of our system share on average 20% of their coexisting strains, while within the set of stable steady states, this overlapping index is nearly always 0. This shows that in multi‐stability, the attractors contain mostly disjoint sets of strains

With the richness of potential dynamics in mind for a fixed μ (Figure 3), we next revisit the relationship between complexity and ratio of single to co‐colonization, with model simulations where $\mu =1/(k\left(R_{0}‐1\right))$ is varied. Using 100,000 random system simulations with N=10 for different values of μ, we confirm the pattern observed for N=2 and illustrated for N=4 in Figure 2, that increasing μ (via decreasing either $R_{0}$ or k) increases strain turnover and the complexity of multi‐strain coexistence. Computing the number and stability properties of equilibria for each system, we can see that multi‐stability of steady‐states is very common, especially in the limit of single colonization dominance (μ→0) as shown in Figure 4a. By multi‐stability we broadly refer to multiple “alternative stable steady‐states” in the system, which may contain different strains, a number of which may also overlap. Pure multi‐stability, with 100% overlapping constituent strains, but different frequencies, between two steady states, is impossible. In contrast, as μ increases, multi‐stable equilibria become less common but the proportion of systems without any stable steady state increases, pointing to an increase in dynamic complexity for the same N and normalized interaction matrix A between strains.

In particular, when zooming‐in on the alternative stable steady‐states of each system, we can quantify how different are the subsets of strains coexisting in each of those equilibria. Defining an index of strain overlap, based on the Jaccard index between two sets (see Appendix), we find that multi‐stability is largely characterized by nonoverlapping subsets of strains (Figure 4b), in contrast to the higher average strain overlap (about 20%) between any two generic steady states. This means that depending on initial conditions and founder effects, the same global pool of N strains with random co‐colonization interactions, can lead to entirely different community composition of strains in different epidemiological settings, an effect that is rather common (60% probability) for values of μ around 0.1, but becomes improbable for μ=10.

**FIGURE 5:**
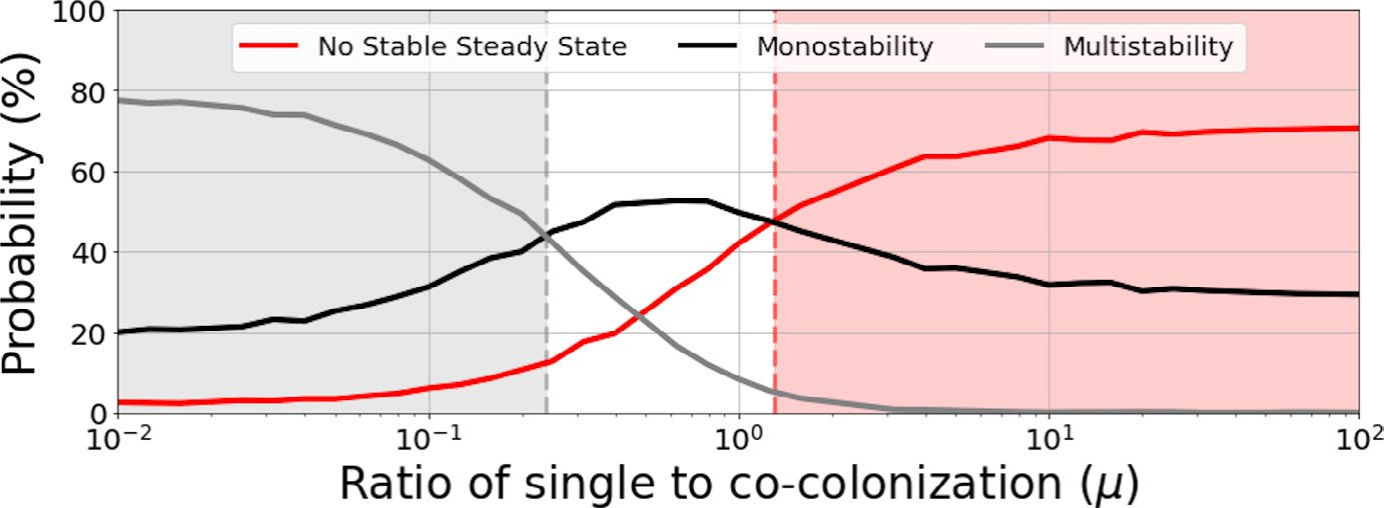
Dynamic regimes as a function of single to co‐colonization ratio μ. We plot the number of steady states as a function of μ∈[0.01,100] for many randomly generated interaction matrices A. Since μ depends on $R_{0}$ and k, we kept their combination implicit, and randomly sampled the rescaled interactions matrix $A=(\alpha_{\mathrm{ij}})$. For any system (A), if there are two or more stable steady states, the system is considered multistable and the dynamics depend on the initial conditions. If there is only one stable steady state the dynamics can be considered simpler. Indeed every system with a unique global attractor is monostable, but the converse is not true for a monostable system may also have non stationary attractors. If there is no steady state, the dynamics are more complex, meaning the attractors are necessarily neither stationary like limit cycles, heteroclinic cycles or even strange attractors. For the simulations, we used a total strain pool size of N=10, and for each value of μ we generated randomly 1,000 matrices A leading to 1,000 different interaction systems for each value of μ. For a given value of μ, we then counted the proportion of systems with no stable steady state (red line), exactly one stable steady state (black line) or several stable steady states (gray line). For a small μ, very few systems have zero stable steady states, while nearly 80% of them are multistable. For a large value of μ the multistability is nearly impossible while more than 70% of interaction systems having no steady state and thus displaying a complex dynamics with no stationary attractors

When quantifying system outcomes for N strains, in three broad categories: those leading to a unique stable steady state, “monostability”; those having multiple alternative stable steady states, “multistability,” and those leading to complex attractors but no stable steady state, as we apply an even higher resolution for μ, we find that there are effectively three regions as a function of single to co‐colonization prevalence (Figure 5). For small μ, stable coexistence and multi‐stability are more likely, for intermediate μ, a single stable steady state is more likely, and for high μ, it is very rare for the system to have any stable steady state, a regime we refer to as *no stable steady state*, implying complexity. In this regime, rock‐paper‐scissors type coexistence and unstable coexistence are the more, if not the only, probable outcomes. This result highlights the importance of an environmental mean‐field gradient in shifting the qualitative dynamics: moving from higher endemic prevalence or more facilitation in co‐colonization towards lower prevalence or more competition, the inter‐dependence between strains increases, the role of asymmetric investment in co‐colonization increases, and thus, the propensity for complex high‐dimensional coexistence becomes higher. In one extreme, we expect low variability in a system over time, while in the other, we expect high variability and complex strain turnover over time.

### 3.4 Contrasting patterns of evolution along the gradient μ

Until now, we have seen how for a fixed μ and a given set of interaction coefficients between strains, their relative abundances change at the population level. Next, we consider how the epidemiological dynamics and the evolutionary dynamics interact. Evolution happens on several levels in this system, made explicit by $z_{i}$ dynamics and various possible trait definitions for each system member i. Here we focus on two important levels. First, frequency dynamics ($z_{i}$) can be linked with selective dynamics on mean interaction in co‐colonization trait space (see Madec & Gjini, 2020) where *k*_effective_ = *k* + *εq*(*t*). Secondly, another trait changing in the community is mean invasibility of the system, Q, in mutual invasion fitness space (see Box 1). These two quantities reflect the changing mean fitness landscape, and depend on frequency dynamics as quadratic terms involving summation over products of strain pair frequencies over time. When varying μ, as the multi‐strain selective dynamics unfold in different ways, so do the evolutionary dynamics of mean traits in the system (Box 3).

BOX 3 **Stability–diversity–complexity and evolutionary dynamics in the system**

In multi‐strain communities with random co‐colonization interaction strengths (A), the global epidemiological quantity $\mu =1/(\left(R_{0}‐1\right)k)$, describing the single to co‐colonization ratio in the host population, modulates regimes of system behavior, shaping its stability, complexity and diversity properties.

Alongside transmission dynamics among hosts, we may track evolution in real time on at least two traits in this system:

1. mean interaction in co‐colonization trait space (*q*) where *k*_effective_ = *k* + *εq*(*t*),
2. mean invasibility of the system (*Q*) in mutual invasion fitness space among strains (Equation 4).

Evolutionary dynamics in the microbial community reflect the changing mean fitness landscape, and depend on strain frequency dynamics (Madec & Gjini, 2020) as follows:

$$q\left(z\right)=\underset{1\leq k,\;j\leq N}{\sum \sum }\alpha_{\mathrm{jk}}z_{k}z_{j}\;\mathrm{and}\;Q\left(z\right)=\underset{1\leq k,\;j\leq N}{\sum \sum }\lambda_{j}^{k}z_{j}z_{k}=\bar{\lambda }.$$

Notice that *q* increasing means the system tends to increase facilitation in co‐colonization on average over time, whereas *q* decreasing means the multi‐strain community tends to increase inhibition in co‐colonization over time. Similarly *Q* increasing implies the system becomes less invadable over time, and *Q* decreasing means the community of strains becomes easier to invade from outsider strains. As frequency dynamics can be complex, so can mean trait dynamics. An indirect measure of the diversity of the system is given by the magnitude of *Q*. A dynamic measure of the “selfishness” in the system is given by the difference:

$$q‐Q=\sum_{i}\alpha_{\mathrm{ii}}z_{i},$$

which indicates the relative strength in the system of self‐positive feedbacks in co‐colonization. If q‐Q>0 the system selects for strains which are better at favoring self than others, and viceversa. If q‐Q<0, the system selects for strains which, on average, are better at favoring others than self (“altruists”). A special case are competitive exclusion scenarios (Madec & Gjini, 2020), where a decrease in Q (Q→0) will be associated with a positive dynamics of q, illustrating how extreme selfishness leads to selection of a single strain in the system.

**Table:** 

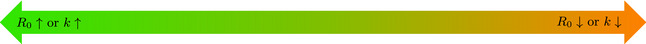	
High prevalence or high facilitation (μ low)	Low prevalence or high competition (μ high)
Co‐colonization dominates Few strains coexist Multistability is common Simple coexistence (fixed point) Low variability over time in one population Mean trait evolution on 2 levels: q and Q over time 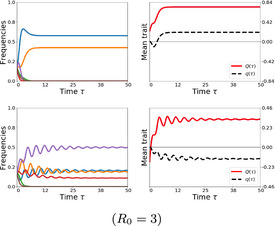 Resistance to invasion Q tends to be stable Either competitors or facilitators can be selected Evolution of “specialist” communities High diversity between host populations	Single colonization dominates Many strains coexist No multi‐stable steady states Complex attractors (limit or heteroclinic cycles) High variability over time in one population Mean trait evolution on 2 levels: q and Q over time 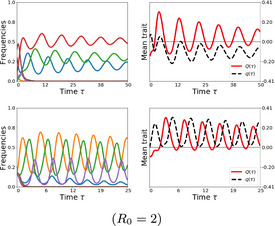 Resistance to invasion Q tends to oscillate Competition and facilitation fluctuate Evolution of “generalist” communities Lower diversity between host populations

In particular, in the μ→0 limit, where multi‐stability is common, systems can slowly evolve towards higher mean competition (q<0), or higher facilitation (q>0) in co‐colonization depending on initial conditions, and on the strains that get selected in the pruned community to stably coexist. Conversely, in the μ→∞ limit, where complex oscillatory coexistence is more probable, systems are more likely to preserve many strains in oscillatory dynamics, and thus fluctuating polymorphism in interaction trait space, with alternating periods between higher average competition and higher average facilitation (q oscillatory). This result highlights two routes to coexistence or maintenance of biodiversity: little variability within one population but diversity between populations in the first case, and more variability within one population but low diversity between populations in the second. Notice also that an increase in mean resistance to invasion of the multi‐strain system (Q dynamics) does not necessarily always correlate with increased facilitation or competition (higher or lower q), and in the oscillatory regime the two can also be synchronous and asynchronous (Figure S6). As q‐Q can be taken as a relative measure of selfishness versus altruism in the system (Box 3), in the asynchronous oscillatory regimes for high μ, we can expect fluctuating prevalence of “altruist” and “selfish” strains, all maintained over time.

### 3.5 Linking μ to the stress gradient hypothesis

Our main result is that the ratio of single to co‐colonization $\mu =1/(\left(R_{0}‐1\right)k)$ is a key modulator of the qualitative complexity of the dynamics (Figure 5, Box 2). While both transmission intensity $R_{0}$ and mean interaction coefficient in cocolonization k decrease this ratio, a natural corollary is that the only way for μ to be held constant, is if $R_{0}$ and k are traded‐off against each other. In other words, when colonization opportunities for all strains are reduced, in order to keep μ constant, the mean interaction coefficient must increase, thus enhancing facilitation among strains in co‐colonization. And viceversa, if the environment gets more favorable, that is, $R_{0}$ increases, then μ can only be be kept constant if k decreases accordingly, thus if all strains become less vulnerable to co‐colonization. Such quantitative feature of our model makes an explicit link with the stress‐gradient hypothesis (SGH) postulated in ecology (Bertness & Callaway, 1994; Callaway & Walker, 1997; Chamberlain et al., 2014). The SGH predicts that positive interactions should be more prevalent in stressful environments, while more favorable environments should favor competition. This hypothesis has been supported and tested mainly in plant systems (Callaway et al., 2002; Eränen & Kozlov, 2008; He et al., 2013; Pugnaire & Luque, 2001), and more recently in microbial communities (Fetzer et al., 2015; Hoek et al., 2016; Lawrence & Barraclough, 2016; McCluney et al., 2012; Piccardi et al., 2019).

**FIGURE 6:**
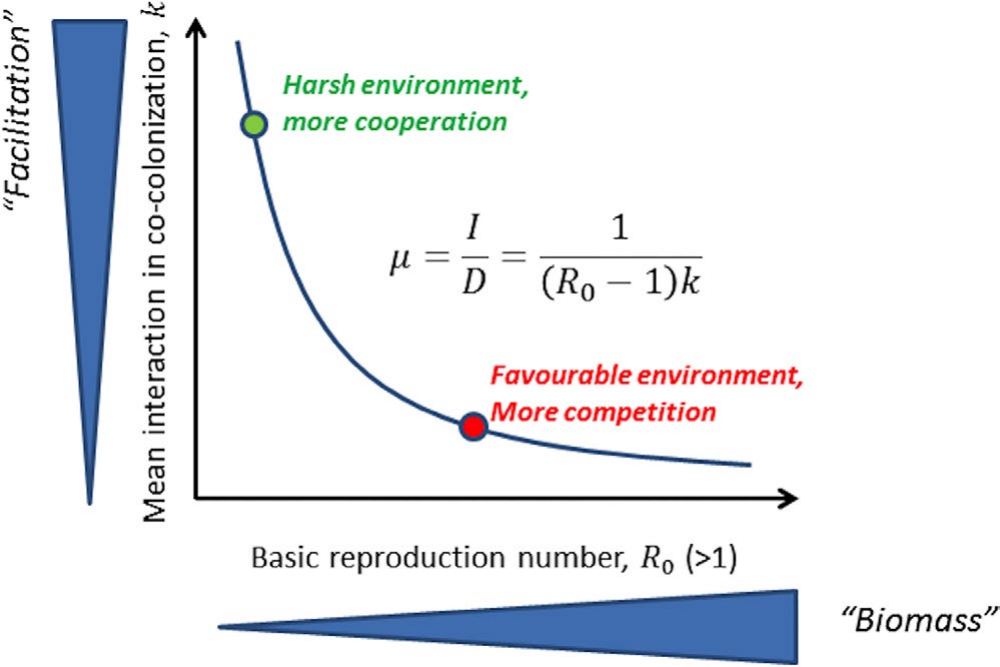
The $R_{0}$ versus k trade‐off in μ and the stress gradient hypothesis. While the type of equilibrium the system will tend to, is strongly driven by μ (the ratio of single to co‐colonization), hence directly by $R_{0}$ and k, it is clear from the expression $\mu =1/(\left(R_{0}‐1\right)k)$ that the only way for this ratio to be held constant, is if $R_{0}$ and k are traded‐off against one another. This principle reflects a core expectation of the stress‐gradient hypothesis (SGH): when transmission opportunities for all strains are reduced (harsher environment), the mean interaction coefficient in the system must increase, that is, if all strains cooperate more in co‐colonization, to keep the same μ and coexistence regime. And viceversa, if the environment gets more favorable, that is, $R_{0}$ increases, then μ can be kept constant only if k decreases, thus if all strains increase competition. Note that keeping μ fixed only preserves the qualitative multi‐strain dynamics, but does not guarantee its speed Θ will remain unchanged. Different global $R_{0}$ and k and the underlying route to such changes may effectively impact selection speed

In our epidemiological system, with microbial interactions embedded in co‐colonization dynamics, the overarching single‐to‐co‐colonization ratio can be considered as a conceptual and mathematical formalization of the stress‐gradient hypothesis. In order for a set of species with a fixed set of normalized interactions (*A* = ($\alpha_{\mathrm{ij}}$)) between them, to maintain a given configuration of coexistence (μ) under changing environmental conditions (e.g., $R_{0}$), the only way is by universal adaptation in mean‐field interaction, increasing antagonism towards co‐colonizers in favorable environments and increasing facilitation in harsher environments, respectively (see Figure 6). However, even if μ is kept constant, the speed of collective dynamics may still be affected, typically reduced with $R_{0}$, although the absolute magnitude will depend on whether and how they are mediated through transmission rate versus duration of carriage (see Figures S3 and S4). In summary, while the fitness landscape is shaped by the phenotypic distribution of all the community members, as the external environment changes, the relative balance of their interactions may shift, due to ultimate context‐dependence in mutual invasion fitness. One solution to keep that balance in check would be by quick reversal adaptation of the mean.

Taken together, our results show that even though differences in pairwise co‐colonization interactions can be small and random, as expected for closely related strains (or species), they are enough to drive selection in a large community of such members, which can collectively coexist, adapt, and self‐organize, albeit often in complex and unstable fashion. The critical influence of average growth potential and mean “willingness to share” co‐colonization, analyzed here, highlights the importance of global context, and calls for detailed system‐specific investigations in the future.

## 4 DISCUSSION

A central question in microbial ecology is whether community members compete or cooperate with one another, and how such interactions mediate community stability, resilience and function. In our model, we study an epidemiological multi‐strain system, where members interact with each other via altered susceptibilities to co‐colonization, which broadly include both competition and facilitation. By clearly delineating the role of mean interaction coefficient, basic reproduction number $R_{0}$, and biases in pairwise coefficients relative to the mean, we obtain a model reduction for strain frequency evolution for any N. The questions we addressed within such a system are similar to a long‐standing ecological quest on multispecies dynamics (Bunin, 2017; May, 1972; Pascual et al., 2006; Serván et al., 2018; Song & Saavedra, 2018): what governs coexistence regimes, diversity and stability in such systems?

Although we do not specify the molecular mechanisms that can mediate positive or negative interactions between strains in co‐colonization (Dawid et al., 2007; Leggett et al., 2014; Lysenko et al., 2010; Riley & Gordon, 1999; Shen et al., 2019), this renders the system generic and broadly applicable. We can quantitatively predict very important features of system behavior with a simple mathematical framework derived under quasi‐neutrality and strain similarity assumptions (Madec & Gjini, 2020). This model reduction captures selective dynamics between strains over long time, and coincides with an instance of the replicator equation from evolutionary game theory (Hofbauer & Sigmund, 2003; Nowak & Sigmund, 2004). Studying this equation and its biological consequences in detail, here we find that in our system, there is a critical ratio that tunes complexity and dynamic regimes in such multi‐strain contagion context: namely the ratio of single to co‐colonization. This ratio, μ, is given by the inverse of the product of the basic reproduction number and mean vulnerability to co‐colonization $\mu =1/(\left(R_{0}‐1\right)k)$. We show that it amplifies the importance of asymmetry in interaction between strains, lending theoretical support to the principle of context‐dependence (Coyte & Rakoff‐Nahoum, 2019) of relative fitnesses in a coupled microbial community.

As strain relative abundances fluctuate, the resource and fitness landscape change dramatically and feed back on the system. This dynamic interdependence is on one hand, the source of complexity, but also a key driver of evolving mean fitness between extant strains. A global, symmetric and temporally varying, environmental feedback on all strains emerges naturally in co‐colonization dynamics (Q in Equation 4), describing mean invasibility and following the evolution of diversity. Mean invasion fitness will tend to increase when more strains are stably maintained over time, and it will tend to zero in the extreme cases of competitive exclusion. Depending on parameter values, there is a gradient for number of resources in the system. Strains compete more strongly for susceptible hosts (one limiting resource) in the extreme of co‐colonization (μ→0), whereas they compete for (at most) N singly colonized hosts, open to co‐colonization (N resources) in the opposite extreme of single colonization dominance (μ→∞).

We find that for small values of the ratio between single and co‐colonization μ, which means high transmission intensity or high cooperation among strains on average, the system dynamics is characterized by multi‐stability, where typically nonoverlapping strain subsets can coexist, depending on initial conditions, a result that resonates with earlier multi‐strain SIRS models with cross‐immunity (Gupta & Anderson, 1999). In our model, we find a similar principle applies, despite the interactions between strains being mediated via altered susceptibilities to co‐colonization, without persistent immune memory. While in the present setup this gradient emerges naturally from the intrinsic structure of infection‐mediated interactions among all strains, similar gradients have been found also in ecological communities studied with generalized Lotka–Volterra models, when the correlation between i‐j and j‐i interactions was explicitly varied (Bunin, 2017).

Our study brings together several themes of interest across many multi‐type systems shaped by interactions and higher‐order feedbacks between their members. Many fields including ecology, geophysics, and economics are calling attention on critical transitions, which occur when natural systems drastically shift from one state to another (Scheffer et al., 2012). Critical transitions in the epidemiology of infectious diseases are of relevance to the emergence of new pathogens and escape from control, such as vaccines. The critical transitions analyzed in this paper relate global and mean‐field environmental variables to the manifestation of competitive hierarchies between multiple strains interacting in co‐colonization. We have made explicit how a gradient emerges from the epidemiological ratio of single to co‐colonization, and how it tunes effectively the diversity, stability and complexity of the coexistence between strains. Such gradient can mediate critical transitions in collective dynamics, when the normalized interaction coefficients between members are held fixed. These transitions may underlie and potentially enhance (or counteract) efforts to control and eliminate multi‐type infectious pathogens, as via vaccines or drugs, or in the face of climate change. Other studies have shown that concurrent multiple infection in malaria creates tipping points that give rise to hysteresis in responses to control or seasonal variation in vector abundance (Alonso et al., 2019). Our work supports a similar perspective, but more generally relevant to interacting systems with multiple strains, and coexistence regimes, rather than prevalence tipping points. Mean facilitation and competition among strains, affecting μ, appear as two sides along a continuum for the system, which particular strain compositions or environmental drivers (seasonality, general host immunity, population turnover) may tip towards one or the other extreme.

We find that when μ tends to favor co‐colonization, for example in the limit of more facilitation between strains on average, the system tends to multi‐stability and stable coexistence of a few strains in simple dynamics. In contrast, when μ tends to favor single colonization, for example in the limit of more competition between strains on average, the system tends to more complexity and unstable equilibria (Figure 5), but coexistence of more strains becomes possible (Figure S1). Although at first sight this may seem to suggest, somewhat contrary to previous expectation from lower‐dimensional models (Chen et al., 2017; Hébert‐Dufresne & Althouse, 2015), that average cooperation in co‐colonization is stabilizing and average competition is destabilizing, our result should be related to the fact that k in our model is not a measure of cooperation in the classical sense, whereby cooperation is exclusively defined as a between‐strain phenomenon. In our model k includes self and nonself‐interaction, and is critical to the global resource dynamics shaped dynamically by N strains.

Our study has also several limitations. Although we explored the qualitative aspects of the dynamics, including the complexity and stability of steady states, their dimensionality and associated entropy, we did not study *which* strains ultimately coexist. This question relates to optimal strategies among N players in a game theoretic context. How the interaction traits of each member determine its persistence or exclusion from the system, and what is the role of N, requires further investigation and algorithmic optimization (Bárány et al., 2007). We also did not develop all the links with the Lotka–Volterra modeling literature in microbial ecology, although special cases of our model are similar to particular cases of GLV dynamics. The metaphor of co‐colonization, as adopted here, could be applied to more general cases of ecological dynamics between microbial species or ecotypes. We did not study alternative distributions of rescaled interaction strengths A, only focusing on a symmetric distribution around 0. It would be interesting in the future to test our findings against nonrandom topologies, and empirical interaction networks, as studied for example by Grilli, Adorisio, et al. (2017). In the limit μ→∞ the patterns analyzed here still hold independently of the distribution governing $A_{\mathrm{ij}}$, but in the limit μ→0, the distribution of A matters, and different distributions may lead to slightly different numerical predictions.

Finally, this model makes several predictions which can be tested empirically. First, the invariant principles in the slow time scale (Box 1) suggest that dominance patterns in single and co‐colonization of particular strains should be the same. The other finding that stable (multi‐stable) coexistence between types through co‐colonization is more likely when mean interactions tend towards cooperation, and that a single stable coexistence between types becomes more probable at intermediate values of μ, and ultimately only unstable coexistence is possible for large values of μ could be tested in endemic multi‐type microbial ecosystems. For example, empirical data in polymorphic *Streptococcus pneumoniae* bacteria, have been consistent with estimates of about 90% mutual inhibition between co‐colonizing serotypes (Gjini et al., 2016; Lipsitch et al., 2012), and $R_{0}$ values around 2. This implies that for this system, μ≈10, and multi‐stability is highly unlikely but a single stable equilibrium point is almost as likely as complex unstable coexistence. This may reconcile the secular trends observed in some settings consistently over many years (Ekdahl et al., 1998; Feikin & Klugman, 2002; Fenoll et al., 1998). Such secular trends can interfere with vaccine introductions and need to be accounted for when estimating impact (Moore, 2009; Moore & Whitney, 2008). Our model makes explicit predictions about the timescale and qualitative aspects of such secular trends (Box 2, Figure S4), under the plausible assumption that they are driven by co‐colonization interactions.

The requirement of being simultaneously stable and feasible tends to push coexistence regimes toward intermediate entropy, independently of precise strain composition. The consistency of the optimal evenness (Shannon entropy) configuration for a given number of strains has been observed empirically for pneumococcus serotypes across geographical settings, before and after vaccination (Hanage et al., 2010). Our model, offers a new perspective on such observations, thanks to co‐colonization interactions between serotypes. As this optimal rank‐order abundance distribution depends on the context μ, model expectations for such dependence could be tested with multi‐site data from different endemicity levels.

We postulate that the stress gradient hypothesis (SGH) (Bertness & Callaway, 1994; Callaway & Walker, 1997; Chamberlain et al., 2014) could help interpret the critical role of multiple infection in shaping epidemiological multi‐strain systems. Our link suggests that to preserve a certain “optimal” single‐to co‐colonization ratio (optimal complexity/coexistence balance), independently of community size N, facilitation in co‐colonization between microbial strains should be more common in settings with low prevalence, a prediction to be tested in the future. It becomes intriguing to verify, beyond pneumococcus, to what extent this model and its insights (Box 3) can be used as an analytic backbone to interpret multi‐strain dynamics in other systems of relevance for public health, for example influenza (Yang et al., 2019), dengue (Mier‐y Teran‐Romero et al., 2013), malaria (Alonso et al., 2019; Gupta & Maiden, 2001) or human papilloma viruses (Murall et al., 2014).

Disentangling multi‐strain interactions and their role in community function at the epidemiological level remains challenging, but can be made more accessible analytically using frameworks such as the one proposed here. With the simplicity and deep insights afforded by this model, we can address better the role of mean fitness of the microbial system as a whole, trait variance, and the role of environmental gradients for stabilizing versus equalizing forces in biodiversity.

## CONFLICT OF INTEREST

The authors declare no conflict of interest.

## AUTHOR CONTRIBUTIONS

**Erida Gjini:** Conceptualization (lead); Data curation (supporting); Formal analysis (supporting); Funding acquisition (equal); Investigation (equal); Methodology (equal); Project administration (lead); Software (supporting); Visualization (supporting); Writing‐original draft (lead); Writing‐review & editing (lead). **Sten Madec:** Conceptualization (supporting); Data curation (lead); Formal analysis (lead); Funding acquisition (equal); Investigation (equal); Methodology (equal); Project administration (supporting); Software (lead); Visualization (lead); Writing‐original draft (supporting); Writing‐review & editing (supporting).

## Supporting information

Supplementary Material

## Data Availability

Illustrative scripts for numerical simulation of the model are available on GitHub: https://github.com/stenmadec/Replicator‐equation‐for‐co‐colonization.

## APPENDIX 1

### 1.1 Strain overlap index (SOI) for two coexistence equilibria of the same system

Consider a set $S=\{E_{1},E_{2},\cdots ,E_{p}\}$ with all steady states of a given system (interaction matrix), where $E_{j}\subset \left\{1,\cdots ,N\right\}$ and $E_{j}\neq \emptyset$. In our applications, $E_{j}$ is the index of feasible strains (with $z_{i}>0$) in the steady‐state number j of a given system of, for example, N=10 strains. We note P(S) the set of each subsets of S. For instance, if $S=\{E_{1},E_{2}\}$ then $P\left(S\right)=\left\{\emptyset ,\left\{E_{1}\right\},\left\{E_{2}\right\},\left\{E_{1},E_{2}\right\}\right\}.$ Recall that $\left|P\left(S\right)\right|=2^{\left|S\right|}=2^{p}$.

We denote the strain overlapping index by SOI and define it as a mean over the Jaccard indices calculated for each pair of coexistence equilibria:

(A.1) $$\text{SOI}=\frac{1}{\left(\begin{array}{c} 2\\ p \end{array}\right)}\sum_{\begin{array}{c} A\in P\left(S\right)\\ \left|A\right|=2 \end{array}}\frac{\left|\underset{E\in A}{⋂}E\right|}{\left|\underset{E\in A}{⋃}E\right|}.$$

### 1.2 Steady states and stability

We give here the mathematical details that have been used for the numerical computation of the steady states of our system and their stability.

#### 1.2.1 Notations

Our replicator equation for strain frequencies (Box 1) reads

(A.2) $${\dot{z}}_{i}=\Theta z_{i}\left({\left(\Lambda z\right)}_{i}‐Q\left(z\right)\right):=f_{i}\left(z\right),\;i=1,\cdots ,N,$$

where $z\left(0\right)\in R_{+}^{N},Q\left(z\right)=z^{T}\Lambda z\;\mathrm{and}\sum_{i=1}^{N}z_{i}\left(0\right)=1.$ In this section, Θ is a positive fixed constant that can be taken equal to one without loss of generality. Define $M=\{z\in R^{N},\sum_{i=1}^{N}z_{i}=1\}$ and the simplex $M_{+}=\left\{z\in R_{+}^{N},\sum_{i=1}^{N}z_{i}=1\right\}=M\cap R_{+}^{N}$. As any replicator equation, $M_{+}$ is invariant under the dynamics of (A.2). Indeed both $R_{+}^{N}$ and M are invariant under (A.2).

#### 1.2.2 Computation of the steady state with n strains

Take a positive integer n≥N and take E⊂{1,⋯,N} with cardinal |E|=n. Denote $\Lambda_{E}\in R^{n\times n}$ the submatrix of Λ with only those columns and those rows whose index belongs to E.

Let $z^{*}$ be a steady state of (A.2) such that $\left\{\begin{array}{c} z_{i}^{*}=0\;\text{if}\;i\;\notin \;E\\ z_{i}^{*}>0\;\text{if}\;i\;\in \;E \end{array}\right)$. Note $z_{E}^{*}={\left(z_{i}\right)}_{i\in E}$. Therefore we have

$$\Lambda_{E}z_{E}^{*}=Q\left(z_{E}^{*}\right){\left(1,\ldots ,1\right)}^{T},\;\sum_{i\in E}z_{i}^{*}=1.$$

In particular, we have ${\left(\Lambda_{E}z_{E}^{*}\right)}_{j}={\left(\Lambda_{E}z_{E}^{*}\right)}_{k}$ for any j,k∈E. This leads to the linear system that we use in the numerical computations:

(A.3) $$\left\{\begin{array}{c} {\left(\Lambda_{E}z_{E}^{*}\right)}_{j}={\left(\Lambda_{E}z_{E}^{*}\right)}_{j+1},\;j=1,\cdots ,n‐1\\ \sum_{i\in E}z_{i}^{*}=1 \end{array}\right).$$

#### 1.2.3 Stability

Denote $f\left(z\right)={\left(f_{i}\left(z\right)\right)}_{i=1,\cdots ,N}$. If $z^{*}$ is a steady state ($f(z^{*})=0$) then the Jacobian matrix of f at $z^{*}$, that we denote $D_{f}\left(z^{*}\right)$, contains all the information about the linear stability of $z^{*}$. But since the dynamics hold only on $M_{+}=M\cap R_{+}^{N}$, the eigenvalue in the direction *transverse* to $M_{+}$ must be removed.

Generally speaking, saying that M is invariant under the dynamics means that for any z∈M, $f\left(z\right)\in T_{z}$ the tangent space of M at z. Moreover, $M=\frac{1}{N}1+M_{0}$ is an affine space where we have denoted $1={\left(1,\cdots ,1\right)}^{T}\in R^{N}$ and $M_{0}=1^{⊥}=\left\{z\in R^{n},z\cdot 1=0\right\}$. Thus, for any z∈M we have $T_{z}=M_{0}$ does not depend on z and thus $f\left(M\right)\subset M_{0}$.

Hence, we define the map $g=f_{|M}:M\to M_{0}$ by g(z)=f(z) for any z∈M. The system (A.2) writes simply as: $\dot{z}=g\left(z\right)$. The stability of a steady state $z^{*}\in M_{+}$ is given by the eigenvalues of the Jacobian $D_{g}\left(z^{*}\right)$ of *g* at $z^{*}$. In practice, the problem is to compute the matrix of this Linear map from the matrix of $D_{f}\left(z^{*}\right)$.

A first idea is to remark that the invariance below implies that $1^{T}$ is a left eigenvector of the Jacobian matrix $J_{f}$ of f at $z^{*}\in M^{+}$ and that the corresponding eigenvalue is $‐Q(z^{*})$. Thus, $‐Q(z^{*})$ is exactly the eigenvalue corresponding to the transverse direction. Hence, we have to remove this eigenvalue from the spectrum of $J_{f}$ to obtain only the eigenvalues of $D_{g}\left(z^{*}\right)$. Due to numerical issues and the possible multiplicity greater than 1 of this eigenvalue, it is hard to use this remark in practice.

Thus we use the following method. Define the vectors $v_{1}=1$ and for $i=1,\ldots ,N,v_{i}\left(j\right)=\left\{\begin{array}{cc} 1 & \text{if}\;j=1\\ ‐1 & \text{if}\;j=i\\ 0 & \text{otherwise} \end{array}\right)$.

The family ${\left\{v_{i}\right\}}_{i=1,\cdots ,N}$ is a base of $R^{N}$ and the subfamily $F_{M}={\left\{v_{i}\right\}}_{i=2,\cdots ,N}$ is a basis of $M_{0}$. Define the matrix $P=(v_{1}|\cdots |v_{n})$.

Let $J_{f}$ be the Jacobian matrix of f at some z in the canonical base. We have

$$P^{‐1}J_{f}P=\left(\begin{array}{cc} ‐Q(z^{*}) & 0_{N‐1}^{T}\\ {}* & J_{g} \end{array}\right),$$

where $0_{n‐1}^{T}$ is a (line) vector with N‐1 null term and ∗ is a (column) vector of $R^{N‐1}$ and $J_{g}$ is the Jacobian matrix of g in the base $F_{M}$. Finally, we compute the stability of $z^{*}$ by the analysis of the spectrum of $J_{g}$.

## References

[ece37259-bib-0001] Adler, F. R. , & Brunet, R. C. (1991). The dynamics of simultaneous infections with altered susceptibilities. Theoretical Population Biology, 40(3), 369–410. 1808757 10.1016/0040-5809(91)90061-j

[ece37259-bib-0002] Alizon, S. , de Roode, J. C. , & Michalakis, Y. (2013). Multiple infections and the evolution of virulence. Ecology Letters, 16(4), 556–567. 23347009 10.1111/ele.12076

[ece37259-bib-0003] Allesina, S. , & Levine, J. M. (2011). A competitive network theory of species diversity. Proceedings of the National Academy of Sciences USA, 108(14), 5638–5642. 10.1073/pnas.1014428108 PMC3078357 21415368

[ece37259-bib-0004] Alonso, D. , Dobson, A. , & Pascual, M. (2019). Critical transitions in malaria transmission models are consistently generated by superinfection. Philosophical Transactions of the Royal Society B: Biological Sciences, 374(1775), 20180275. 10.1098/rstb.2018.0275 PMC6553601 31056048

[ece37259-bib-0005] Bárány, I. , Vempala, S. , & Vetta, A. (2007). Nash equilibria in random games. Random Structures & Algorithms, 31(4), 391–405.

[ece37259-bib-0006] Bascompte, J. (2019). Mutualism and biodiversity. Current Biology, 29(11), R467–R470. 31163160 10.1016/j.cub.2019.03.062

[ece37259-bib-0007] Bertness, M. D. , & Callaway, R. (1994). Positive interactions in communities. Trends in Ecology & Evolution, 9(5), 191–193. 21236818 10.1016/0169-5347(94)90088-4

[ece37259-bib-0008] Bomze, I. M. (1995). Lotka‐volterra equation and replicator dynamics: New issues in classification. Biological Cybernetics, 72(5), 447–453.

[ece37259-bib-0009] Bucci, V. , Tzen, B. , Li, N. , Simmons, M. , Tanoue, T. , Bogart, E. , Deng, L. , Yeliseyev, V. , Delaney, M. L. , Liu, Q. , Olle, B. , Stein, R. R. , Honda, K. , Bry, L. , & Gerber, G. K. (2016). Mdsine: Microbial dynamical systems inference engine for microbiome time‐series analyses. Genome Biology, 17(1), 121. 27259475 10.1186/s13059-016-0980-6 PMC4893271

[ece37259-bib-0010] Bunin, G. (2017). Ecological communities with lotka‐volterra dynamics. Physical Review E, 95(4), 042414. 28505745 10.1103/PhysRevE.95.042414

[ece37259-bib-0011] Callaway, R. M. , Brooker, R. , Choler, P. , Kikvidze, Z. , Lortie, C. J. , Michalet, R. , Paolini, L. , Pugnaire, F. I. , Newingham, B. , Aschehoug, E. T. , Armas, C. , Kikodze, D. , & Cook, B. J. (2002). Positive interactions among alpine plants increase with stress. Nature, 417(6891), 844–848. 12075350 10.1038/nature00812

[ece37259-bib-0012] Callaway, R. M. , & Walker, L. R. (1997). Competition and facilitation: A synthetic approach to interactions in plant communities. Ecology, 78(7), 1958–1965.

[ece37259-bib-0013] Chamberlain, S. A. , Bronstein, J. L. , & Rudgers, J. A. (2014). How context dependent are species interactions? Ecology Letters, 17(7), 881–890. 24735225 10.1111/ele.12279

[ece37259-bib-0014] Chawanya, T. , & Tokita, K. (2002). Large‐dimensional replicator equations with antisymmetric random interactions. Journal of the Physical Society of Japan, 71(2), 429–431.

[ece37259-bib-0015] Chen, L. , Ghanbarnejad, F. , & Brockmann, D. (2017). Fundamental properties of cooperative contagion processes. New Journal of Physics, 19(10), 103041.

[ece37259-bib-0016] Cobey, S. , & Lipsitch, M. (2012). Niche and neutral effects of acquired immunity permit coexistence of pneumococcal serotypes. Science (New York, NY), 335, 1376–1380. 10.1126/science.1215947 PMC3341938 22383809

[ece37259-bib-0017] Coyte, K. Z. , & Rakoff‐Nahoum, S. (2019). Understanding competition and cooperation within the mammalian gut microbiome. Current Biology, 29(11), R538–R544. 31163167 10.1016/j.cub.2019.04.017 PMC6935513

[ece37259-bib-0018] Cressman, R. , & Tao, Y. (2014). The replicator equation and other game dynamics. Proceedings of the National Academy of Sciences USA, 111(Suppl 3), 10810–10817. 10.1073/pnas.1400823111 PMC4113915 25024202

[ece37259-bib-0019] Davies, N. G. , Flasche, S. , Jit, M. , & Atkins, K. E. (2019). Within‐host dynamics shape antibiotic resistance in commensal bacteria. Nature Ecology & Evolution, 3(3), 440. 30742105 10.1038/s41559-018-0786-x PMC6420107

[ece37259-bib-0020] Dawid, S. , Roche, A. M. , & Weiser, J. N. (2007). The blp bacteriocins of *Streptococcus pneumoniae* mediate intraspecies competition both in vitro and in vivo. Infection and Immunity, 75(1), 443–451. 17074857 10.1128/IAI.01775-05 PMC1828380

[ece37259-bib-0021] Diekmann, O. , Heesterbeek, J. , & Metz, J. A. (1990). On the definition and the computation of the basic reproduction ratio R0 in models for infectious diseases in heterogeneous populations. Journal of Mathematical Biology, 28(4), 365–382. 2117040 10.1007/BF00178324

[ece37259-bib-0022] Ekdahl, K. , Mårtensson, A. , & Kamme, C. (1998). Bacteraemic pneumococcal infections in southern sweden 1981–96: Trends in incidence, mortality, age‐distribution, serogroups and penicillin‐resistance. Scandinavian Journal of Infectious Diseases, 30(3), 257–262. 9790133 10.1080/00365549850160891

[ece37259-bib-0023] Eränen, J. K. , & Kozlov, M. V. (2008). Increasing intraspecific facilitation in exposed environments: Consistent results from mountain birch populations in two subarctic stress gradients. Oikos, 117(10), 1569–1577.

[ece37259-bib-0024] Feikin, D. R. , & Klugman, K. P. (2002). Historical changes in pneumococcal serogroup distribution: Implications for the era of pneumococcal conjugate vaccines. Clinical Infectious Diseases, 35(5), 547–555. 12173128 10.1086/341896

[ece37259-bib-0025] Fenoll, A. , Jado, I. , Vicioso, D. , Pérez, A. , & Casal, J. (1998). Evolution of *Streptococcus pneumoniae* serotypes and antibiotic resistance in Spain: Update (1990 to 1996). Journal of Clinical Microbiology, 36(12), 3447–3454. 9817852 10.1128/jcm.36.12.3447-3454.1998 PMC105219

[ece37259-bib-0026] Fetzer, I. , Johst, K. , Schäwe, R. , Banitz, T. , Harms, H. , & Chatzinotas, A. (2015). The extent of functional redundancy changes as species? Roles shift in different environments. Proceedings of the National Academy of Sciences USA, 112(48), 14888–14893. 10.1073/pnas.1505587112 PMC4672811 26578806

[ece37259-bib-0027] Fisher, D. C. , & Reeves, R. B. (1995). Optimal strategies for random tournament games. Linear Algebra and Its Applications, 217, 83–85.Proceedings of a Conference on Graphs and Matrices in Honor of John Maybee.

[ece37259-bib-0028] Friedman, J. , Higgins, L. M. , & Gore, J. (2017). Community structure follows simple assembly rules in microbial microcosms. Nature Ecology & Evolution, 1(5), 0109. 10.1038/s41559-017-0109 28812687

[ece37259-bib-0029] Gaivão, M. , Dionisio, F. , & Gjini, E. (2017). Transmission fitness in co‐colonization and the persistence of bacterial pathogens. Bulletin of Mathematical Biology, 79(9), 2068–2087. 28741105 10.1007/s11538-017-0320-3

[ece37259-bib-0030] Gjini, E. , & Madec, S. (2017). A slow‐fast dynamic decomposition links neutral and non‐neutral coexistence in interacting multi‐strain pathogens. Theoretical Ecology, 10(1), 129–141.

[ece37259-bib-0031] Gjini, E. , Valente, C. , Sá‐Leão, R. , & Gomes, M. G. M. (2016). How direct competition shapes coexistence and vaccine effects in multi‐strain pathogen systems. Journal of Theoretical Biology, 388, 50–60. 26471070 10.1016/j.jtbi.2015.09.031

[ece37259-bib-0032] Gog, J. R. , & Grenfell, B. T. (2002). Dynamics and selection of many‐strain pathogens. Proceedings of the National Academy of Sciences USA, 99(26), 17209–17214. 10.1073/pnas.252512799 PMC139294 12481034

[ece37259-bib-0033] Gomes, M. G. M. , Medley, G. F. , & Nokes, D. J. (2002). On the determinants of population structure in antigenically diverse pathogens. Proceedings of the Royal Society of London B: Biological Sciences, 269(1488), 227–233. 10.1098/rspb.2001.1869 PMC1690892 11839191

[ece37259-bib-0034] Grilli, J. , Adorisio, M. , Suweis, S. , Barabás, G. , Banavar, J. R. , Allesina, S. , & Maritan, A. (2017). Feasibility and coexistence of large ecological communities. Nature Communications, 8, 14389. 10.1038/ncomms14389 PMC5333123 28233768

[ece37259-bib-0035] Grilli, J. , Barabás, G. , Michalska‐Smith, M. J. , & Allesina, S. (2017). Higher‐order interactions stabilize dynamics in competitive network models. Nature, 548(7666), 210–213. 10.1038/nature23273 28746307

[ece37259-bib-0036] Gupta, S. , & Anderson, R. (1999). Population structure of pathogens: The role of immune selection. Parasitology Today, 15(12), 497–501. 10557151 10.1016/s0169-4758(99)01559-8

[ece37259-bib-0037] Gupta, S. , Ferguson, N. , & Anderson, R. (1998). Chaos, persistence, and evolution of strain structure in antigenically diverse infectious agents. Science, 280(5365), 912–915. 9572737 10.1126/science.280.5365.912

[ece37259-bib-0038] Gupta, S. , & Maiden, M. C. (2001). Exploring the evolution of diversity in pathogen populations. Trends in Microbiology, 9(4), 181–185. 11286883 10.1016/s0966-842x(01)01986-2

[ece37259-bib-0039] Hanage, W. P. , Finkelstein, J. A. , Huang, S. S. , Pelton, S. I. , Stevenson, A. E. , Kleinman, K. , Hinrichsen, V. L. , & Fraser, C. (2010). Evidence that pneumococcal serotype replacement in Massachusetts following conjugate vaccination is now complete. Epidemics, 2(2), 80–84. 21031138 10.1016/j.epidem.2010.03.005 PMC2963072

[ece37259-bib-0040] He, Q. , Bertness, M. D. , & Altieri, A. H. (2013). Global shifts towards positive species interactions with increasing environmental stress. Ecology Letters, 16(5), 695–706. 23363430 10.1111/ele.12080

[ece37259-bib-0041] Hébert‐Dufresne, L. , & Althouse, B. M. (2015). Complex dynamics of synergistic coinfections on realistically clustered networks. Proceedings of the National Academy of Sciences USA, 112(33), 10551–10556. 10.1073/pnas.1507820112 PMC4547298 26195773

[ece37259-bib-0042] Hoek, T. A. , Axelrod, K. , Biancalani, T. , Yurtsev, E. A. , Liu, J. , & Gore, J. (2016). Resource availability modulates the cooperative and competitive nature of a microbial cross‐feeding mutualism. PLoS Biology, 14(8), e1002540. 10.1371/journal.pbio.1002540 27557335 PMC4996419

[ece37259-bib-0043] Hofbauer, J. , & Sigmund, K. (2003). Evolutionary game dynamics. Bulletin of the American Mathematical Society, 40(4), 479–519.

[ece37259-bib-0044] Ke, P.‐J. , & Letten, A. D. (2018). Coexistence theory and the frequency‐dependence of priority effects. Nature Ecology & Evolution, 2(11), 1691–1695. 30297744 10.1038/s41559-018-0679-z

[ece37259-bib-0045] Kerr, B. , Riley, M. A. , Feldman, M. W. , & Bohannan, B. J. (2002). Local dispersal promotes biodiversity in a real‐life game of rock–paper–scissors. Nature, 418(6894), 171–174. 12110887 10.1038/nature00823

[ece37259-bib-0046] Landi, P. , Minoarivelo, H. O. , Brännström, Å. , Hui, C. , & Dieckmann, U. (2018). Complexity and stability of ecological networks: A review of the theory. Population Ecology, 60(4), 319–345.

[ece37259-bib-0047] Law, R. , & Morton, R. D. (1996). Permanence and the assembly of ecological communities. Ecology, 77(3), 762–775.

[ece37259-bib-0048] Lawrence, D. , & Barraclough, T. G. (2016). Evolution of resource use along a gradient of stress leads to increased facilitation. Oikos, 125(9), 1284–1295.

[ece37259-bib-0049] Leggett, H. C. , Brown, S. P. , & Reece, S. E. (2014). War and peace: Social interactions in infections. Philosophical Transactions of the Royal Society B: Biological Sciences, 369(1642), 20130365. 10.1098/rstb.2013.0365 PMC3982666 24686936

[ece37259-bib-0050] Levine, J. M. , Bascompte, J. , Adler, P. B. , & Allesina, S. (2017). Beyond pairwise mechanisms of species coexistence in complex communities. Nature, 546(7656), 56–64. 28569813 10.1038/nature22898

[ece37259-bib-0051] Lin, J. , Andreasen, V. , & Levin, S. A. (1999). Dynamics of influenza a drift: The linear three‐strain model. Mathematical Biosciences, 162(1–2), 33–51. 10616279 10.1016/s0025-5564(99)00042-5

[ece37259-bib-0052] Lipsitch, M. (1997). Vaccination against colonizing bacteria with multiple serotypes. Proceedings of the National Academy of Sciences USA, 94(12), 6571–6576. 10.1073/pnas.94.12.6571 PMC21091 9177259

[ece37259-bib-0053] Lipsitch, M. , Abdullahi, O. , D'Amour, A. , Xie, W. , Weinberger, D. M. , Tchetgen, E. T. , & Scott, J. A. G. (2012). Estimating rates of carriage acquisition and clearance and competitive ability for pneumococcal serotypes in Kenya with a Markov transition model. Epidemiology, 23(4), 510–519. 22441543 10.1097/EDE.0b013e31824f2f32 PMC3670084

[ece37259-bib-0054] Lipsitch, M. , Colijn, C. , Cohen, T. , Hanage, W. P. , & Fraser, C. (2009). No coexistence for free: Neutral null models for multistrain pathogens. Epidemics, 1(1), 2–13. 21352747 10.1016/j.epidem.2008.07.001 PMC3099423

[ece37259-bib-0055] Lotka, A. J. (1926). Elements of physical biology. Science Progress in the Twentieth Century (1919–1933), 21(82), 341–343.

[ece37259-bib-0056] Lysenko, E. S. , Lijek, R. S. , Brown, S. P. , & Weiser, J. N. (2010). Within‐host competition drives selection for the capsule virulence determinant of *Streptococcus pneumoniae* . Current Biology, 20(13), 1222–1226. 20619820 10.1016/j.cub.2010.05.051 PMC2913241

[ece37259-bib-0057] Madec, S. , & Gjini, E. (2020). Predicting n‐strain coexistence from co‐colonization interactions: Epidemiology meets ecology and the replicator equation. Bulletin of Mathematical Biology, 82(11), 142. 10.1007/s11538-020-00816-w 33119836 PMC7595998

[ece37259-bib-0058] May, R. M. (1972). Will a large complex system be stable? Nature, 238(5364), 413–414. 10.1038/238413a0 4559589

[ece37259-bib-0059] McCann, K. S. (2000). The diversity–stability debate. Nature, 405(6783), 228–233. 10821283 10.1038/35012234

[ece37259-bib-0060] McCluney, K. E. , Belnap, J. , Collins, S. L. , González, A. L. , Hagen, E. M. , Nathaniel Holland, J. , Kotler, B. P. , Maestre, F. T. , Smith, S. D. , & Wolf, B. O. (2012). Shifting species interactions in terrestrial dryland ecosystems under altered water availability and climate change. Biological Reviews, 87(3), 563–582. 22098619 10.1111/j.1469-185X.2011.00209.x

[ece37259-bib-0061] Mier‐y Teran‐Romero, L. , Schwartz, I. B. , & Cummings, D. A. (2013). Breaking the symmetry: Immune enhancement increases persistence of dengue viruses in the presence of asymmetric transmission rates. Journal of Theoretical Biology, 332, 203–210. 23665358 10.1016/j.jtbi.2013.04.036 PMC3782297

[ece37259-bib-0062] Momeni, B. , Xie, L. , & Shou, W. (2017). Lotka‐volterra pairwise modeling fails to capture diverse pairwise microbial interactions. Elife, 6, e25051. 28350295 10.7554/eLife.25051 PMC5469619

[ece37259-bib-0063] Moore, M. R. (2009). Rethinking replacement and resistance. Journal of Infectious Diseases, 199(6), 771–773. 10.1086/597045 19434926

[ece37259-bib-0064] Moore, M. R. , & Whitney, C. G. (2008). Emergence of nonvaccine serotypes following introduction of pneumococcal conjugate vaccine: Cause and effect? Clinical Infectious Diseases, 46(2), 183–185. 10.1086/524661 18171248

[ece37259-bib-0065] Morrison, K. E. (2013). From bocce to positivity: Some probabilistic linear algebra. Mathematics Magazine, 86(2), 110–119.

[ece37259-bib-0066] Mosquera, J. , & Adler, F. R. (1998). Evolution of virulence: A unified framework for coinfection and superinfection. Journal of Theoretical Biology, 195(3), 293–313. 9826485 10.1006/jtbi.1998.0793

[ece37259-bib-0067] Mougi, A. , & Kondoh, M. (2012). Diversity of interaction types and ecological community stability. Science, 337(6092), 349–351. 22822151 10.1126/science.1220529

[ece37259-bib-0068] Murall, C. L. , McCann, K. S. , & Bauch, C. T. (2014). Revising ecological assumptions about human papillomavirus interactions and type replacement. Journal of Theoretical Biology, 350, 98–109. 24412334 10.1016/j.jtbi.2013.12.028

[ece37259-bib-0069] Nowak, M. A. , & Sigmund, K. (2004). Evolutionary dynamics of biological games. Science, 303(5659), 793–799. 14764867 10.1126/science.1093411

[ece37259-bib-0070] Odum, E. P. , & Barrett, G. W. (1971). Fundamentals of ecology (Vol. 3). Saunders.

[ece37259-bib-0071] Pascual, M. , Dunne, J. A. , Dunne, J. A. , (Eds.). (2006). Ecological networks: Linking structure to dynamics in food webs. Oxford University Press.

[ece37259-bib-0072] Piccardi, P. , Vessman, B. , & Mitri, S. (2019). Toxicity drives facilitation between 4 bacterial species. Proceedings of the National Academy of Sciences USA, 116(32), 15979–15984. 10.1073/pnas.1906172116 PMC6690002 31270235

[ece37259-bib-0073] Pugnaire, F. I. , & Luque, M. T. (2001). Changes in plant interactions along a gradient of environmental stress. Oikos, 93(1), 42–49.

[ece37259-bib-0074] Riley, M. A. , & Gordon, D. M. (1999). The ecological role of bacteriocins in bacterial competition. Trends in Microbiology, 7(3), 129–133. 10203843 10.1016/s0966-842x(99)01459-6

[ece37259-bib-0075] Scheffer, M. , Carpenter, S. R. , Lenton, T. M. , Bascompte, J. , Brock, W. , Dakos, V. , Van de Koppel, J. , Van de Leemput, I. A. , Levin, S. A. , Van Nes, E. H. , Pascual, M. , & Vandermeer, J. (2012). Anticipating critical transitions. Science, 338(6105), 344–348. 23087241 10.1126/science.1225244

[ece37259-bib-0076] Serván, C. A. , Capitán, J. A. , Grilli, J. , Morrison, K. E. , & Allesina, S. (2018). Coexistence of many species in random ecosystems. Nature Ecology & Evolution, 2(8), 1237. 29988167 10.1038/s41559-018-0603-6

[ece37259-bib-0077] Shen, P. , Lees, J. A. , Bee, G. C. W. , Brown, S. P. , & Weiser, J. N. (2019). Pneumococcal quorum sensing drives an asymmetric owner–intruder competitive strategy during carriage via the competence regulon. Nature Microbiology, 4(1), 198–208. 10.1038/s41564-018-0314-4 PMC6342471 30546100

[ece37259-bib-0078] Song, C. , & Saavedra, S. (2018). Will a small randomly assembled community be feasible and stable? Ecology, 99(3), 743–751. 10.1002/ecy.2125 29285752

[ece37259-bib-0079] Stein, R. R. , Bucci, V. , Toussaint, N. C. , Buffie, C. G. , Rätsch, G. , Pamer, E. G. , Sander, C. , & Xavier, J. B. (2013). Ecological modeling from time‐series inference: Insight into dynamics and stability of intestinal microbiota. PLoS Computational Biology, 9(12), e1003388. 24348232 10.1371/journal.pcbi.1003388 PMC3861043

[ece37259-bib-0080] Tilman, D. , & Downing, J. A. (1994). Biodiversity and stability in grasslands. Nature, 367(6461), 363–365.

[ece37259-bib-0081] van Baalen, M. , & Sabelis, M. W. (1995). The dynamics of multiple infection and the evolution of virulence. American Naturalist, 146, 881–910. 10.1086/285830

[ece37259-bib-0082] Volterra, V. (1926). Fluctuations in the Abundance of a Species considered Mathematically. Nature, 118, 558–560. 10.1038/118558a0

[ece37259-bib-0083] Wearing, H. J. , & Rohani, P. (2006). Ecological and immunological determinants of dengue epidemics. Proceedings of the National Academy of Sciences USA, 103(31), 11802–11807. 10.1073/pnas.0602960103 PMC1544250 16868086

[ece37259-bib-0084] Yang, W. , Lau, E. H. , & Cowling, B. J. (2019). Dynamic interactions of influenza viruses in Hong Kong during 1998–2018. medRxiv, 19008987. 10.1371/journal.pcbi.1007989 PMC7316359 32542015

[ece37259-bib-0085] Yoshino, Y. , Galla, T. , & Tokita, K. (2008). Rank abundance relations in evolutionary dynamics of random replicators. Physical Review E, 78, 031924. 10.1103/PhysRevE.78.031924 18851082

