# Supplementary material for: The ratio of single to co‐colonization is key to complexity in interacting systems with multiple strains
Source: Ecol Evol. 2021 Jun 6;11(13):8456–74. doi: 10.1002/ece3.7259 (PMC8258234; doi:10.1002/ece3.7259)
Supplement: Supplementary file 1 — Supplementary Material [file ECE3-11-8456-s001.pdf]

Supporting information

The ratio of single to co-colonization is key to complexity in interacting systems with multiple strains

Erida Gjini[1,2*] and Sten Madec[3**]

[1]Instituto Gulbenkian de Ciência, Oeiras, Portugal
[2]CEMAT, Instituto Superior Tecnico, University of Lisbon, Lisbon, Portugal
[3]Institut Denis Poisson, University of Tours, France
[*]erida.gjini@tecnico.ulisboa.pt
[**]Sten.Madec@univ-tours.fr

S1 Supplementary figures

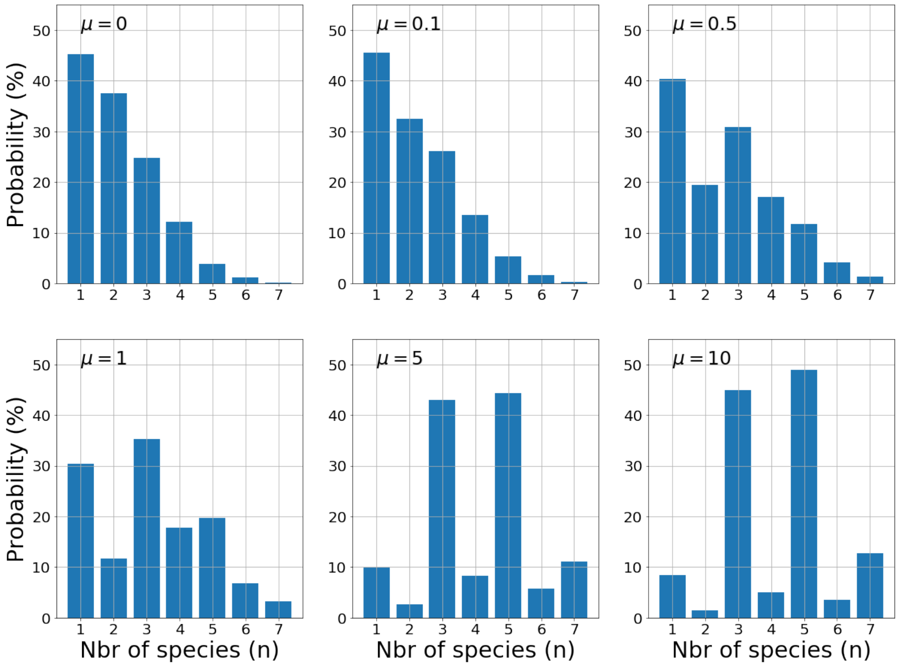

Figure S1: **Probability for stable n-strain coexistence out of a pool of $N = 10$ strains.**We plot the distribution of the number of coexisting strains n for 6 values of μ. For each value of μ, we generated 100 000 matrices A (normalized interactions between strains) and computed their stable steady states. The data are the same as those in the figures 3 and 4. We find that the distribution of n is very dependent on μ. In general, the probability is hard to compute explicitly but the limit $\mu \to \infty$ is explicitly known using the fact that the pairwise invasion fitness matrix becomes skew symmetric in that limit (see Box 2). One remarkable fact is that for small μ the probability of finding a stable steady state is greater for $n = 1$ and decreases very quickly with number of strains n, while for large values of n, the distribution looks like a binomial distribution centered at $\frac{N}{2}$ for odd values of n, and zero for even values of n. Hence, the probability to have a large number of strains coexisting at a given steady state is small for small μ and increases for large values of μ. In particular, for $\mu \in \{0, 0.1, 0.5, 1, 5, 10\}$, the mean number $E[n]$ of coexisting strains increases with μ and is respectively $E[n] = 2.16,\ 2.26,\ 2.68,\ 3.17,\ 4.14,\ 4.21$.Finally, it can be computed that $E[n] \to \frac{N}{2} = 5$ as $\mu \to \infty$.

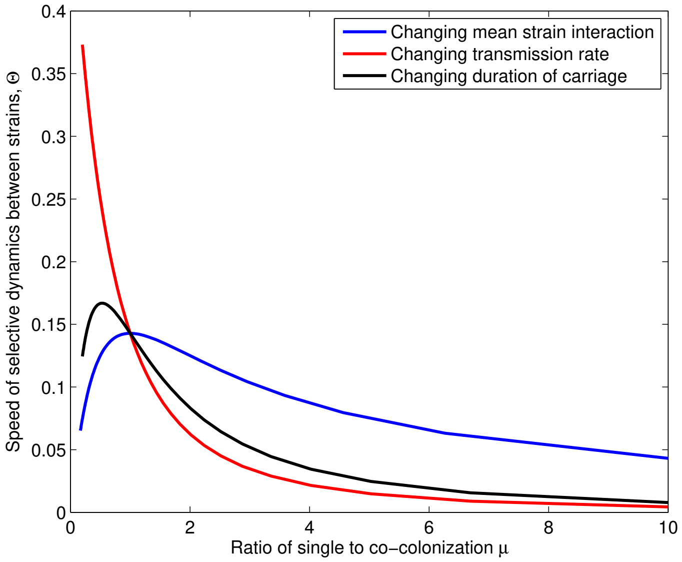

Figure S2: **Speed of dynamics Θ depends on μ and other parameters.** As derived in (Madec and Gjini, 2020), $\Theta = \beta(1 - \frac{1}{R_0})(\frac{\mu}{2(\mu+1)^2-\mu})$. The ratio of single to co-colonization μ can vary in three different ways. When k is varied: $\beta = 2, m = 1, R_0 = 2$, μ decreases with k, but Θ first increases then decreases with μ (blue line). When R_0 is varied: via β: $k = 1, m = 1$, μ decreases with R_0 and Θ decreases with μ (red line). When R_0 is varied via m: $k = 1, \beta = 1$, μ again decreases with R_0, while Θ increases, then decreases with μ (black line). The effects of the same change in μ on the parameter Θ are different for these three different cases.

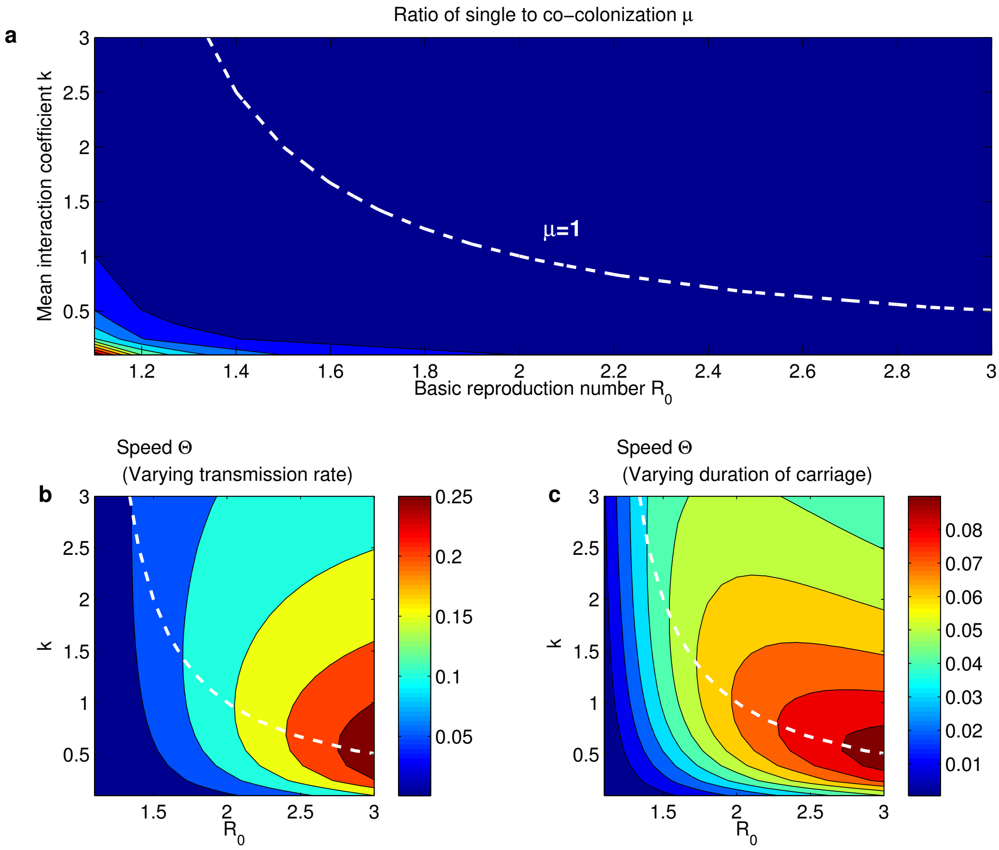

Figure S3: **Keeping μ constant via the trade-off $R_0 - k$ can have different quantitative effects on the speed of the dynamics depending on epidemiological drivers.** a. Relationship between R_0 and k in determining μ. b) Assuming fixed $m = 1$, $\beta = R_0 m$ is varied to give rise to variation in a). c) Assuming fixed $\beta = 1$, $m = R_0/\beta$ is varied to give rise to variation in a). The same change in μ can lead to different effects on Θ in these two cases.

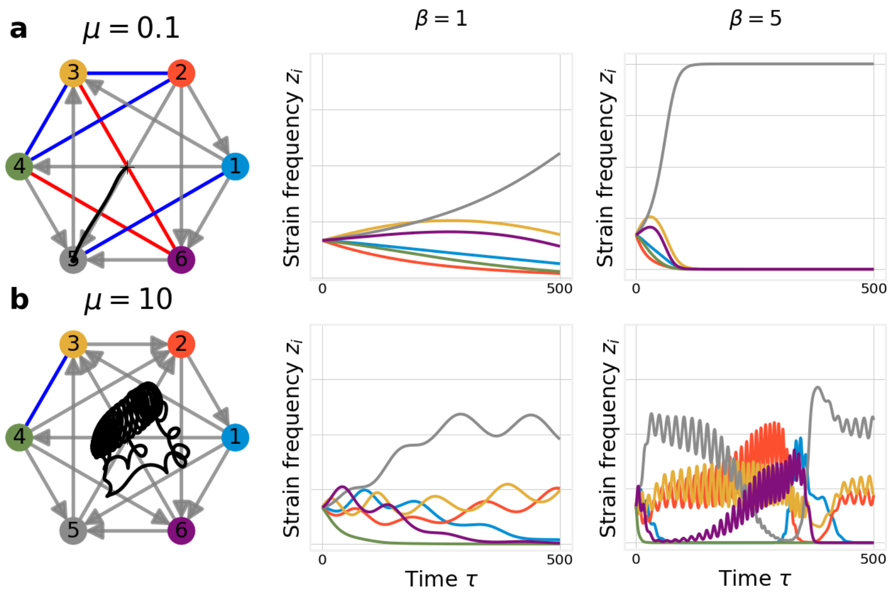

Figure S4: **Keeping μ constant preserves the same qualitative dynamics in principle but there are timescale effects.** For two values of μ (rows a and b) we compute the dynamics and we show the multi-strain frequency trajectories for two different choice of transmission rate β (columns 2 and 3). μ and β being fixed, we have $k = 1/\mu(R_0 - 1)$. For the normalized matrix of interactions A we assume the same matrix in each figure

$$A = \begin{pmatrix} -0.1 & -0.5 & 0.5 & -0.3 & -0.1 & -0.1 \\ -0.1 & -0.5 & 0.2 & -0.3 & -0.3 & -0.4 \\ -0.2 & -0.3 & 0.2 & -0.4 & 0.2 & 0.5 \\ -0.4 & -0.4 & 0. & 0.3 & -0.1 & 0.4 \\ -0. & -0. & 0. & -0.1 & 0.4 & -0.2 \\ 0.2 & 0.1 & -0.4 & 0.1 & -0. & -0.3 \end{pmatrix}.$$

We assume $m = 0.5$ (clearance plus natural mortality rate) so that $R_0 = 2\beta$ and a maximum time for simulation $T_{max} = 500$. For each row the qualitative dynamics remain the same as shown on the graph. However, the speed of this dynamics depends on β. **a.** On the first row $\mu = 0.1$, we have a competitive exclusion scenario. Only strain 5 persists. When $\beta = 1$ (and then $k = 10$ to compensate via the trade-off for μ constant) all the strains are still present in the system at T_{max}. For $\beta = 5$ (then $k = 100/9$), dynamics are much quicker and selection of finally only one strain occurs very rapidly. The values of Θ are respectively: 0.043, and 0.39. **b.** In the second row $\mu = 10$. For this value of μ, the same matrix A yields more complex dynamics in general, in line with the result that qualitatively the dynamics are determined by μ. However, when $\beta = 2$, the dynamics seems to approach a cycle with dominance of strain 5 (grey) and extinction of strains 1, (blue) 4 (green) and 6 (purple). For $\beta = 10$ (so $k = 1/90$), the dynamics over the same timescale show a very complex behavior. First strain 6 appears to come back, and 5 goes to extinction, then strain 1 comes back again and ultimately strain 5 dominates in coexistence with 2 and 3. The values of Θ respectively are: 0.043, and 0.39. Thus increasing transmission rate has increased the speed about ten fold.

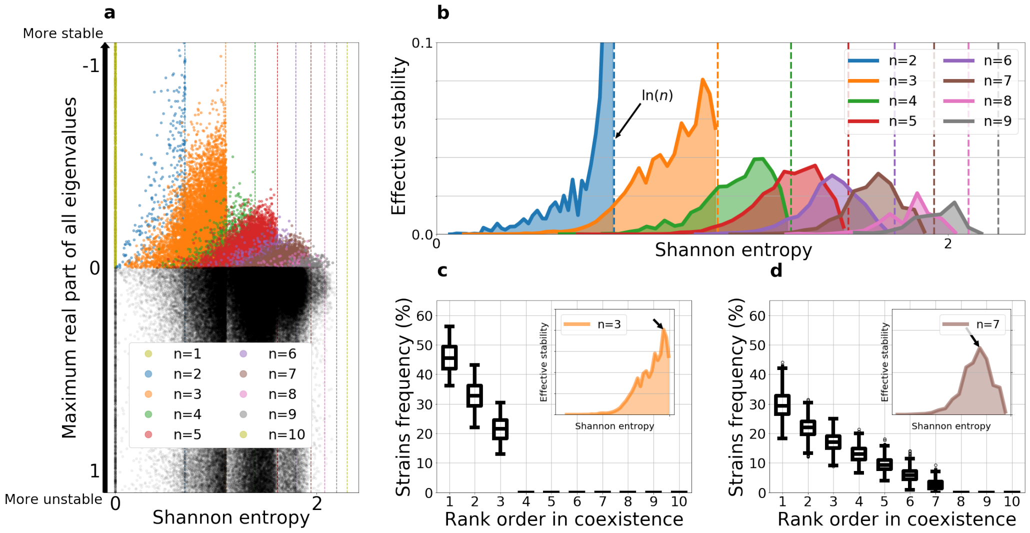

Figure S5: **Diversity-Stability relationship for $\mu = 10$.** This figure is similar to figure 3 but for $\mu = 10$ (which is more realistic for pneumococcus). We repeat the same simulations for $N = 10$. **a.** Relation between Shannon entropy (x-axis) and stability (y-axis) for different number of n coexisting strains, starting with a pool of $N = 10$. Due to this large value of $\mu = 10$, it is very rare to observe an even number of coexisting strains n (see figure S1). **b.** However, the shape of the effective stability continues to be similar to the case $\mu = 0.05$ for both even and odd n. For $n \geq 3$, there is an "optimal evenness" in the community which leads to the optimal balance between feasibility and stability of the multi-strain coexistence steady state. **c. and d.** To be consistent, we illustrate the rank order at the "optimal evenness" for the odd numbers of coexisting species $n = 3$ and $n = 7$.

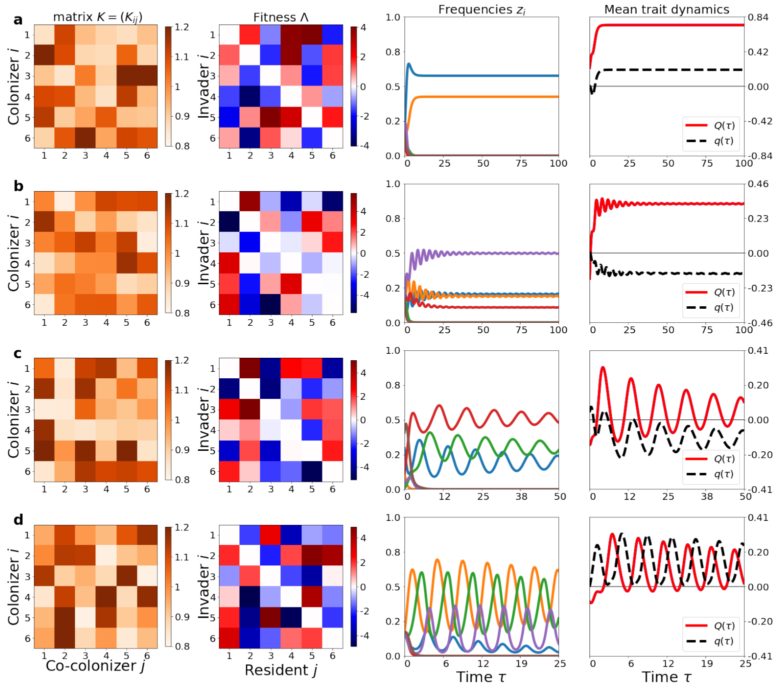

Figure S6: **Evolutionary dynamics of mean traits in the N-strain system.** **a.-b** Two cases of $R_0 = 3$, where the dynamics of q and Q can be correlated or anti-correlated. Evolution of system resilience to invasion (Q high) can be obtained with selection of more facilitation ($q > 0$) or more competition ($q < 0$) in the system, depending on the system and initial conditions. **c-d.** Two cases of $R_0 = 2$, where since μ is higher, the dynamics are more complex, and so is evolution of mean traits in the system. Remarkably q and Q can be synchronous or asynchronous. In c) the difference $q - Q < 0$, suggesting a general selection of 'altruistic' strains in the system. In d) $q - Q$ oscillates around zero, suggesting fluctuating prevalences of 'altruist' and 'selfish' strains in the system. The high μ regime corresponds more likely to maintenance of polymorphism in interaction trait space within the same population.

S2 Mathematical details for the limits of $\mu \to 0$ and $\mu \to \infty$

We use the following notations, for the basic reproduction number R_0, and for the global steady state (S^*, T^*, I^*, D^*) obtained via the neutral system (where $K_{ij} \equiv k$):

$$R_0 = \frac{\beta}{m},\ S^* = \frac{1}{R_0},\ T^* = 1 - S^*,$$

$$I^* = \frac{\beta S^* T^*}{k\beta T^* + m} = \frac{R_0 - 1}{R_0(k(R_0-1)+1)},$$

$$D^* = T^* - I^* = k(R_0 - 1)I^*.$$

We define the important ratio μ for the relative dominance of single over co-colonization by

$$\mu = \frac{I^*}{D^*} = \frac{1}{k(R_0-1)}. \tag{S1}$$

The reduced system for strain frequency dynamics reads

$$\dot{z}_i = \Theta z_i \left((\Lambda z)_i - z^t \Lambda z\right), \quad i = 1, \cdots, N \tag{S2}$$

with the constraint

$$\sum_{i=1}^{N} z_i = 1, \quad z_i \geq 0.$$

The *qualitative* behavior depends on the fitness $N \times N$ matrix $\Lambda = (\lambda_i^j)_{i,j}$ whose i, j term is the fitness of strain i with respect to the strain j and is explicitly given by

$$\lambda_i^j = \mu(\alpha_{j,i} - \alpha_{i,j}) + \alpha_{j,i} - \alpha_{j,j}.$$

Here, $A = (\alpha_{i,j}) = ((K_{ij} - k)/\varepsilon)$ is the normalized interaction matrix, accounting for deviation from neutrality.
Recall that λ_i^j denotes the growth rate of the strain i when introduced to the steady state of the strain j alone : $E_j = (z_1, \cdots, z_N)$ where $z_j = 1$, $z_k = 0$ for $k \neq j$.

The time scale of the selective dynamics between multiple strains, in this system, depends basically on the constant

$$\Theta = \frac{\beta T^* I^* D^*}{2(T^*)^2 - I^* D^*}.$$

Our goal here is to give a description of the system in the limit cases $\mu \to 0$ and $\mu \to +\infty$.
There are 3 parts in the discussion.

(i) Which parameters change? μ depends on k, β and m. This point is straightforward from the formula (S1).

(ii) The qualitative behaviour. This depends only on Λ (up to a positive multiplicative constant) which depends only on μ. Hence the point (i) has no importance here.

(iii) How does the speed of the dynamics $(\Theta\|\Lambda\|)$ vary? This speed is especially important when it is close to 0. Remember that this system has been obtained by focusing of the first order perturbation in the quasi-neutral approximation (the zero order perturbation, $\varepsilon = 0$, yields exactly the neutral model). If the speed of the dynamics is below some threshold $O(\varepsilon)$, then we cannot remove the next order perturbation and our model does not describe well the dynamics. In other words, if the speed is too slow then the system will stay very close to neutrality for a very long time. We will say that the system is *effectively neutral.* Hence, the main question in this section is the following:

Can the speed of dynamics go to zero when μ changes? If yes, what is the relevance of the parameters in point (i)?

S2.1 Limit 1 : Co-colonization, $\mu \to 0$: $k \to +\infty$ or $R_0 \to +\infty$.

The qualitative dynamics. Recall that $\lambda_i^j > 0$ means that the strain i (the invader) may invade the system from a very small concentration within a system where only the strain j (the resident) is present.

Passing to the limit $\mu \to 0$, we obtain the matrix Λ_{lim} with the (i, j) coefficient being simply

$$\lambda_i^j = \alpha_{j,i} - \alpha_{j,j}.$$

In general, very little may be said, because the structure of the matrix Λ_{lim} can be anything. As an example, in the special case $\alpha_{jj} = 0$ for all j, then $\Lambda_{\text{lim}} = A^T$. Since A may have any structure, so can Λ_{lim}, and very few results are known for the general replicator system. However, it is useful to see A as a random matrix and to check the probability of a given type of dynamics (see Yoshino et al. (2008)). We have the following interpretation.

- The fitness λ_i^j depends only on the coefficients $\alpha_{j,i}$ and $\alpha_{j,j}$ which are characteristics of the resident. $\alpha_{j,i}$ measures how much the resident j contributes to the pool of co-colonization by the strains i and j : I_{ij}, whereas $\alpha_{j,j}$ measures how much the resident j increases the pool of self co-colonization: I_{jj}.

- Hence, the relative fitness of the invader i depends only of the resident j. The invader i may invade the resident j if and only if the resident reinforces more the co-colonization I_{ij} than I_{jj}.
- This phenomenon provides a lot of niches for multiple strains to coexist. As an example, if A has a strong diagonal : $\alpha_{j,j} > \alpha_{i,j}$ for any $j = 1, \cdots N$ and $i \neq j$ then all the strains may be stable residents when alone, and we will have at least N stable monomorphic steady states.
- In conclusion, let A be chosen randomly. The system is likely to have a lot of stable steady states wherein only few strains coexist. It is rare to see cycles or more complex dynamics.

The speed of the dynamics. Since $\mu \to 0$ then μ is bounded and far from 0, $\|\Lambda\|$ is bounded and positive. The speed of the dynamics is then measured by Θ which is computed as:

$$\Theta = \frac{\frac{m}{k}}{2(\mu+1)^2 - \mu}. \tag{S3}$$

- If $k \to +\infty$, from the second expression in (S3) we have: $\Theta \sim \frac{m}{2k} \to 0 = \Theta_{\lim}$. This is a problematic situation. *If the second infection occurs very quickly then Θ is very small and the model does not capture the real dynamics.*
- If $m \to 0$ then again the same expression give $\Theta \sim \frac{m}{2k} \to \Theta_{\lim} = 0$. *If the natural mortality or the clearance of infection happen very slowly (which means that the rate of population turnover is very slow) then this model do not capture the real dynamics.*
- If $\beta \to +\infty$ then the same equation above shows that $\Theta \to \frac{m}{2k} = \Theta_{\lim} > 0$ which, in this case, is far from zero. *Thus, if the pathogen transmission rate is very large, then the system captures well the real dynamics and the qualitative study of $\mu \to 0$ is appropriate.*

In summary, the only way for $\Theta_{\lim}$ to be positive as $\mu \to 0$ is when $\beta \to +\infty$.

S2.2 Limit 2 : single colonization, $\mu \to +\infty$: $k \to 0$ or $R_0 \to 1$.

In that cases, we have generically $\|\Lambda\| \to +\infty$ and $\Theta \to 0$. In order to keep a bounded matrix Λ we rewrite the system as

$$\dot{z_i} = \mu\Theta z_i \left(\left(\left(\mu^{-1}\Lambda\right) z\right)_i - z^t \left(\mu^{-1}\Lambda\right) z\right), \quad i = 1, \cdots, N \tag{S4}$$

The speed is then given by $\mu\theta$ and the qualitative behavior by $\mu^{-1}\Lambda$.

The qualitative dynamics. When $\mu \to +\infty$, the matrix $\mu^{-1}\Lambda \to \Lambda_{\lim} = A^T - A$ This matrix of pairwise invasion fitnesses becomes skew symmetric.

As opposed to the previous limit, there exist some results for the skew symmetric case. In the particular case where the coefficients are ± 1 the system is known as the *Tournament game* which has been studied in detail in in Fisher and Reeves (1995) and has been used recently in Allesina and Levine (2011) in coexistence theory. Some of these results remain true for a general skew matrix Chawanya and Tokita (2002).

- The quadratic term in the frequency evolution equation is always[1] zero. So the replicator equation system for N strains reduces to
$$\dot{z_i} = \Theta_{\lim} z_i \left(\left((\Lambda_{\lim}) z\right)_i\right), \quad i = 1, \cdots, N, \quad \sum_{i=1}^{N} z_i = 1. \tag{S5}$$
- Generically, there exists exactly one non-negative linearly stable equilibrium (in particular multistability is impossible). Without loss of generality, we can assume that this equilibrium reads $\zeta = (\zeta_1, \cdots, \zeta_n, 0, \cdots, 0)$ with $1 \leq n \leq N$.
- From the linear point of view, ζ is a center: it is linearly stable but not asymptotically linearly stable. Indeed, the $N - n$ last eigenvalues are negative and the n first eigenvalues are purely imaginary.
- The function $H(z) = \sum_{i=1}^{N} \zeta_i \ln(z_i)$ is bounded and increasing. We have $\dot{H} \to 0$ and $\dot{H}(z) = 0$ if and only $z_k = 0$ for each $k > n$.
- It follows that $z_k \to 0$ for each $k > n$, and that the sub system of the n first strains is strongly persistent.
- In practice, exactly like the classical Lotka-Volterra model of prey and predators, we have a one-parameter family of cycles, parametrized by initial conditions, more precisely by the value of $H(z(0))$.
- Denote $\Lambda_{\lim}^n$ the sub-matrix of the first n rows and n columns of Λ^∞ and $\zeta_n = (\zeta_1, \cdots, \zeta_n)$. We have
$$\Lambda_{\lim}^n \zeta_n = 0$$
In particular it is necessary that $|\Lambda_{\lim}^n| = 0$. Since $\lambda_{\lim}^n$ is skew symmetric, we have
$$|\Lambda_{\lim}^n| = | - \Lambda_{\lim}^n| = (-1)^n |\Lambda_{\lim}^n|.$$
Hence, if n is odd we always have $|\Lambda_{\lim}^n| = 0$. On the contrary, if n is even $\|\Lambda_{\lim}^n\|$ has no reason to be zero (except in very specific cases which have a zero probability of occurrence in the sense below) and there will be no steady state of n coexisting strains.

[1] The quadratic part is the scalar $Q(z) = z^T \Lambda z$. From $\Lambda = -\Lambda$ we obtain $Q(z) = Q(z)^T = z^T \Lambda^T z = -Q(z)$, thus $Q(z) = 0$.

- A more complete description may be given. Let the coefficient of Λ_{lim} be chosen randomly. Denote n the number of strains coexisting, then the probability to have a steady state of $n = k$ strains over a total pool of N strains is

$$P(n=k) = \begin{cases} 0 & \text{if } k \text{ is even} \\ \binom{N}{k} 2^{1-N} & \text{if } k \text{ is odd} \end{cases}$$

We illustrate this case in the figure S1.

We finish this section by highlighting the fact that the above-described dynamics is not structurally stable. This structure will be lost for a large but finite value of μ. As μ decreases from $+\infty$, the center will become either a stable focus or an unstable focus. In the first case, we will observe a global attractor, while in the second case, which is the most probable as shown in Figure 5, we will observe more complex dynamics like heteroclinic cycle or even chaos.

The speed of the dynamics As shown below, the speed, in this second limit, is given by

$$\mu\Theta = \frac{\beta\mu^2\left(1 - \frac{1}{R_0}\right)}{2(\mu+1)^2 - \mu} \tag{S6}$$

- If $R_0 \to 1$ then[2] $\mu\Theta \to 0 = \Theta_{\text{lim}}$ and the model is effectively neutral.
  If the transmission intensity of the infection is too low, then this model do not capture well the dynamics.
- If $k \to 0$ then $\mu\Theta \to \Theta_{\text{lim}} = \frac{m}{2}(R_0 - 1) > 0$ which is far from zero.

In summary : the only way for Θ_{lim} to be positive as $\mu \to +\infty$ is to have $k \to 0$, thus in the limit of extreme competition between strains in co-colonization. In that case, the compartment of co-colonization D becomes very small, and the dynamics are well described by the approximation $\mu \to +\infty$, which depends only on the asymmetries $\alpha_{j,i} - \alpha_{i,j}$.

References

Allesina, S. and Levine, J. M. (2011). A competitive network theory of species diversity. *Proceedings of the National Academy of Sciences*, 108(14):5638–5642.

Chawanya, T. and Tokita, K. (2002). Large-dimensional replicator equations with antisymmetric random interactions. *Journal of the Physical Society of Japan*, 71(2):429–431.

Fisher, D. C. and Reeves, R. B. (1995). Optimal strategies for random tournament games. *Linear Algebra and its Applications*, 217:83 – 85. Proceedings of a Conference on Graphs and Matrices in Honor of John Maybee.

Madec, S. and Gjini, E. (2020). Predicting n-strain coexistence from co-colonization interactions: epidemiology meets ecology and the replicator equation. *Bulletin of Mathematical Biology*, 82(142).

Yoshino, Y., Galla, T., and Tokita, K. (2008). Rank abundance relations in evolutionary dynamics of random replicators. *Phys. Rev. E*, 78:031924.

[2]Except if $\beta \to +\infty$ and $R_0 = \frac{\beta}{m} \to 1$, like by example if $\beta = m + a$. This corresponds to a situation where both the mortality-clearance rate and the infection rate become simultaneously very large. In that case, we rewrite $\mu\Theta\frac{\mu^2(\beta-m)}{2(\mu+1)^2-\mu} \to \theta_{\text{lim}} = \frac{a}{2}$ which is positive.
